# OpenSAFELY: The impact of COVID‐19 on azathioprine, leflunomide and methotrexate monitoring, and factors associated with change in monitoring rate

**DOI:** 10.1111/bcp.16062

**Published:** 2024-04-08

**Authors:** Andrew D. Brown, Louis Fisher, Helen J. Curtis, Milan Wiedemann, William J. Hulme, Victoria Speed, Lisa E. M. Hopcroft, Christine Cunningham, Ruth E. Costello, James B. Galloway, Mark D. Russell, Katie Bechman, Zeyneb Kurt, Richard Croker, Chris Wood, Alex J. Walker, Andrea L. Schaffer, Seb C. J. Bacon, Amir Mehrkar, George Hickman, Chris Bates, Jonathan Cockburn, John Parry, Frank Hester, Sam Harper, Ben Goldacre, The OpenSAFELY Collaborative, Brian MacKenna

**Affiliations:** ^1^ Bennett Institute for Applied Data Science, Nuffield Department of Primary Care Health Sciences University of Oxford UK; ^2^ London School of Hygiene and Tropical Medicine London UK; ^3^ Centre for Rheumatic Diseases King's College London UK; ^4^ Northumbria University Newcastle upon Tyne UK; ^5^ TPP Leeds UK

**Keywords:** antirheumatic agents, azathioprine, COVID‐19, electronic health records, general practice, leflunomide, methotrexate

## Abstract

**Aims:**

The COVID‐19 pandemic created unprecedented pressure on healthcare services. This study investigates whether disease‐modifying antirheumatic drug (DMARD) safety monitoring was affected during the COVID‐19 pandemic.

**Methods:**

A population‐based cohort study was conducted using the OpenSAFELY platform to access electronic health record data from 24.2 million patients registered at general practices using TPP's SystmOne software. Patients were included for further analysis if prescribed azathioprine, leflunomide or methotrexate between November 2019 and July 2022. Outcomes were assessed as monthly trends and variation between various sociodemographic and clinical groups for adherence with standard safety monitoring recommendations.

**Results:**

An acute increase in the rate of missed monitoring occurred across the study population (+12.4 percentage points) when lockdown measures were implemented in March 2020. This increase was more pronounced for some patient groups (70–79 year‐olds: +13.7 percentage points; females: +12.8 percentage points), regions (North West: +17.0 percentage points), medications (leflunomide: +20.7 percentage points) and monitoring tests (blood pressure: +24.5 percentage points). Missed monitoring rates decreased substantially for all groups by July 2022. Consistent differences were observed in overall missed monitoring rates between several groups throughout the study.

**Conclusion:**

DMARD monitoring rates temporarily deteriorated during the COVID‐19 pandemic. Deterioration coincided with the onset of lockdown measures, with monitoring rates recovering rapidly as lockdown measures were eased. Differences observed in monitoring rates between medications, tests, regions and patient groups highlight opportunities to tackle potential inequalities in the provision or uptake of monitoring services. Further research should evaluate the causes of the differences identified between groups.

**What is already known about this subject**

- Disease‐modifying antirheumatic drugs (DMARDs) carry risks of serious adverse effects, so national guidelines recommend that patients taking them adhere to regular safety monitoring.
- The COVID‐19 pandemic disrupted general monitoring services in England, with certain groups disproportionately affected in their health outcomes.

**What this study adds**

- DMARD monitoring rates deteriorated during the COVID‐19 pandemic but recovered quickly after the easing of lockdown measures.
- Substantial variations in DMARD safety monitoring rates were found amongst different demographic, clinical and regional subgroups in England—suggesting that opportunities exist to address inequalities in safety monitoring.

## 1 INTRODUCTION

The COVID‐19 pandemic markedly disrupted delivery of primary care services globally,^1^ and within the National Health Service (NHS), with a notable impact on chronic disease management.^2^ Disease‐modifying antirheumatic drugs (DMARDs) are typically prescribed for chronic autoimmune conditions such as rheumatoid arthritis, Crohn's disease and severe psoriasis. Many DMARDs have narrow therapeutic indexes, and can cause potentially fatal adverse events such as blood dyscrasias and liver toxicity.^3^, ^4^ To mitigate these risks, DMARDs are typically initiated by a specialist clinician in secondary care. Once patients have been stabilized on treatment, they can be transferred to primary care under a shared care arrangement, which specifies long‐term safety monitoring recommendations (Table 1) for general practitioners (GPs) to oversee. Under the arrangement GPs have guidance on when to suspend a prescription or seek specialist advice, e.g. if they observe unsafe monitoring results, nonadherence to monitoring or emergence of adverse effects.

**TABLE 1:** Examples of licensed indications typically seen in primary care, and long‐term monitoring requirements for 3 commonly prescribed disease‐modifying antirheumatic drugs (DMARDs).

Drug name	Indications	Long‐term monitoring
Azathioprine	Crohn's disease, severe eczema, myasthenia gravis.	Every 12 weeks: • Full blood count • Urea and electrolytes • Liver function tests
Leflunomide	Rheumatoid arthritis, psoriatic arthritis.	Every 12 weeks: • Full blood count • Urea and electrolytes • Liver function tests • Blood pressure • Weight
Methotrexate	Crohn's disease, severe psoriasis, rheumatoid arthritis.	Every 12 weeks: • Full blood count • Urea and electrolytes • Liver function tests

The first wave of the COVID‐19 pandemic in March 2020 prompted a national lockdown in the UK. GP practices made several changes with the aim of reducing viral transmission, ranging from reducing face‐to‐face appointments, to temporary practice closure where outbreaks occurred amongst practice staff.^5^ Moreover, many patients worldwide chose to avoid healthcare services for fear of contracting COVID‐19,^6^, ^7^, ^8^ and those who were recommended to isolate through *shielding* faced additional barriers to healthcare access.^9^ In April 2020, the British Medical Association and Royal College of GPs issued guidance suggesting clinicians consider DMARD monitoring a high priority task.^10^ However, The NHS Specialist Pharmacy Service and British Society of Rheumatology suggested that monitoring intervals for certain medications, including DMARDs, could be considered for extension.^11^, ^12^ Several laboratory tests in GP practices experienced substantial activity reductions during lockdown,^13^ including tests related to methotrexate monitoring.^14^ To date, no research has described the extent to which adherence with wider DMARD monitoring recommendations was affected during the COVID‐19 pandemic.

OpenSAFELY is a secure analytics platform for electronic patient records built with the approval of NHS England to deliver urgent academic^15^ and operational NHS service research^13^, ^16^ on the impacts of the pandemic. Analyses run across patients' full raw pseudonymized primary care records at English GP practices with patient‐level linkage to sources of secondary care data.

We set out to assess the impact of COVID‐19 on the monitoring of DMARDs using OpenSAFELY, and evaluate whether any such effect was associated with other variables, such as patient demographics or comorbidities.

## 2 METHODS

### 2.1 Study design

We conducted a retrospective cohort study across 24 million patients by using EHR data from all GP practices in England supplied by software provider TPP. From this data we sought to identify patients prescribed DMARDs between November 2019 and July 2022.

### 2.2 Data source

Primary care records managed by TPP are available in OpenSAFELY, a data analytics platform created by our team on behalf of NHS England to address urgent COVID‐19 research questions (https://opensafely.org). OpenSAFELY provides a secure software interface allowing analysis of pseudonymized primary care patient records from England in near real‐time within the EHR vendor's secure data centre, avoiding the need for large volumes of potentially disclosive pseudonymized patient data to be transferred off‐site. This, in addition to other technical and organizational controls, minimizes risk of re‐identification. Similarly pseudonymized datasets from other data providers are securely provided to the EHR vendor and linked to the primary care data. The dataset analysed within OpenSAFELY is based on people currently registered with GP practices using TPP SystmOne software. It includes pseudonymized data such as coded diagnoses, medications and physiological parameters. No free text data are included. Further details can be found under information governance and ethics.

### 2.3 Study population

We included all patients who were: alive; aged 18–120 years; and registered with a TPP practice. Index of multiple deprivation (IMD)^17^ and rurality classification^18^ were derived for each patient based on their address. We excluded patients missing age, sex, IMD and rurality classification, since their omission may indicate generally poor data quality.

We defined patients on each DMARD as those recorded with at least 2 prescriptions (one issued within 3 months before the search date, and the other between 3 and 6 months before the search date). Three‐month search windows for prescriptions were chosen because in England GPs issue 93% of repeat prescriptions for a duration of 3 months or less.^19^

Azathioprine, leflunomide and methotrexate were chosen as a representative sample of DMARDs. These 3 medications are consistently classified as shared care across England, which avoids introducing potential confounding in the analysis of monitoring rates from inconsistent shared care statuses. We created codelists for each DMARD using NHS dictionary of medicines and devices (dm+d).

### 2.4 Study measures and statistics

Monitoring tests relevant to the prescribed medication were selected (Table 1). These included full blood counts (FBCs), liver function tests (LFTs), urea and electrolytes (U&E), and blood pressure (BP). We excluded weight (for leflunomide) because it is not consistently listed as a monitoring requirement in all sources; namely the British National Formulary and Summary of Product Characteristics. Moreover, BP and weight checks would usually be carried out at the same time, so BP can be considered broadly representative of *physical* health checks.

We selected patient groups which were relevant to health inequality priorities in England,^20^ or deemed susceptible to variation in monitoring. We grouped age into 7 categories (18–29, 30–39, 40–49, 50–59, 60–69, 70–79, ≥80 years), sex into male or female, ethnicity into 5 categories (Black, Mixed, South Asian, White, Other), region into 9 categories (East, East Midlands, London, North East, North West, South East, South West, West Midlands, Yorkshire and The Humber), rural–urban into 8 categories (1: *most urban*, to 8: *most rural*),^18^ and IMD into 5 quintiles. Dementia, learning disability, serious mental illness, care home status and housebound status were grouped as binary characteristics.

Codelists were specified to represent monitoring tests and patient groups using SNOMED CT. Each monitoring test typically represents a set of tests conducted together, therefore a single representative test was chosen (Table S1). For example, where FBCs are recommended we looked for the presence of a Red blood cell count code.

Patients were defined as having missed monitoring if any of the relevant tests required for their given drug were not recorded in the last 3 months. Indicators (see Table S2) expressed the proportion of patients deemed to have missed monitoring (numerator), of all relevant patients prescribed the DMARD(s) being assessed (denominator). Higher percentages represent poorer monitoring performance. Each indicator was specified in analytic code within the OpenSAFELY framework, and was calculated from patient counts rounded to the nearest 5 to ensure anonymity.

#### 2.4.1 Trend in DMARD monitoring rates

The numerator and denominator were generated for each indicator at monthly intervals between November 2019 and July 2022, then percentages calculated for each time‐period. Note that each month in our results reflects a 3‐month rolling average corresponding to data for the named month and the 2 months preceding it. For example, *May 2020* represents patients not having tests from 1 March 2020 to 31 May 2020. Time‐periods of interest are defined in Table 2.

**TABLE 2:** Time‐periods (captured as 3‐month rolling averages) of interest in relation to disease‐modifying antirheumatic drug (DMARD) monitoring during the COVID‐19 pandemic.

Name	Time‐period	Rationale for interest
Baseline period	February 2020	The 3 months prior to March 2020 represent baseline activity as no restrictions had been enforced prior to this and the pandemic was still in an early phase.
Lockdown period	May 2020	The pandemic rapidly escalated during March with National *lockdown* being enforced on 23rd March 2020. All monitoring tests analysed are associated with a routine 3‐monthly monitoring window, e.g. FBC is required to be checked every 3 months. So, the 3 months following the onset of disruption represents the point at which all patients would be subject to delayed monitoring; a *worst case scenario* if no action was taken to rectify COVID‐19 related delays.
Recovery period	July 2022	No significant restrictions were in place at this time. As the most recent month in the study, this represents the closest return to normalcy, and a relevant point to consider adherence to monitoring rates for current policymaking.

Abbreviation: FBC, full blood count.

Total counts of the numerator and denominator were calculated for each indicator across the full study period. In this cumulative data, repeated events were counted for each period in which the event occurs, e.g. if a patient missed monitoring in 2 separate periods this was represented as 2 separate events. To report the extent of monitoring being repeatedly missed for the same patients, we also calculated the ratio of total missed monitoring events to unique patients with missed monitoring events across the full study period.

We conducted *t*‐tests to investigate whether a significant change in monitoring rate occurred between February–May 2020.

#### 2.4.2 Variability of change across GP practices

The indicator percentages for overall DMARD missed monitoring rate were summarized as deciles across all GP practices and presented as a decile chart.

#### 2.4.3 Factors associated with change in monitoring rate

Cochran's Q heterogeneity tests were conducted within each demographic/clinical subgroup to check for associations between patient factors and changes in monitoring rate.

### 2.5 Software and reproducibility

Data management and analysis was performed using the OpenSAFELY software libraries and Python, both implemented using Python 3.8. Inferential statistics were performed using R. This analysis was delivered using federated analysis, which involves carrying out patient‐level analysis in multiple secure datasets, then later combining them: codelists and code for data management and analysis were specified once using the OpenSAFELY tools; then securely transmitted to the OpenSAFELY‐TPP platform for execution against local patient data within TPP's secure environment. Summary results were reviewed for disclosiveness and released for final outputs. All code for the OpenSAFELY platform for data management, analysis and secure code execution is shared for review and re‐use under open licences at github.com/OpenSAFELY, or for this study at github.com/opensafely/Shared‐Care‐Monitoring.

### 2.6 Patient and public involvement

Ensuring patient, professional and public trust is vital. Maintaining trust requires being transparent about how OpenSAFELY works, and ensuring that patient voices are represented when designing research, analysing findings and considering implications. Our website https://opensafely.org/ provides a detailed description of the platform in language suitable for a lay audience. We have participated in citizen juries exploring public trust in OpenSAFELY;^21^ we are currently codeveloping an explainer video; we have *expert by experience* patient representation on our OpenSAFELY Oversight Board; we have partnered with Understanding Patient Data to produce lay explainers on the importance of large datasets for research; we have presented at several public engagement events. We also work closely with appropriate medical research charities.

## 3 RESULTS

Demographic, regional and clinical characteristics of patients being regularly prescribed azathioprine, leflunomide or methotrexate are reported in Table 3 according to the final month of the study period (July 2022). Of these 94 611 patients, 60.9% were aged ≥60 years, 60.9% were female and 91.1% were White.

**TABLE 3:** Cohort description for patients who were included in at least 1 disease‐modifying antirheumatic drug (DMARD) denominator at the end of the study period (July 2022).

Characteristic	Category	*n*	%^a^
Population	Total	94 611	100.0%
Age band	18–29	3465	3.7%
	30–39	5905	6.2%
	40–49	9555	10.1%
	50–59	18 050	19.1%
	60–69	22 410	23.7%
	70–79	23 490	24.8%
	80+	11 730	12.4%
Sex	Female	57 620	60.9%
	Male	36 990	39.1%
Ethnicity	Black	1050	1.1%
	Mixed	625	0.7%
	Other	750	0.8%
	South Asian	5180	5.5%
	White	86 215	91.1%
	Missing	790	0.8%
Region	East	23 105	24.4%
	East Midlands	16 365	17.3%
	London	3040	3.2%
	North East	5040	5.3%
	North West	7860	8.3%
	South East	6605	7.0%
	South West	15 260	16.1%
	West Midlands	2925	3.1%
	Yorkshire and The Humber	14 140	15.0%
	Missing	270	0.3%
Care home	Yes	575	0.6%
	No	94 035	99.4%
Dementia	Yes	1370	1.5%
	No	93 245	98.6%
Housebound	Yes	3380	3.6%
	No	91 235	96.4%
Learning disability	Yes	375	0.4%
	No	94 235	99.6%
Serious mental illness	Yes	940	1.0%
	No	93 670	99.0%
Index of multiple deprivation	1st quintile (most deprived)	15 160	16.0%
	2nd quintile	17 555	18.6%
	3rd quintile	21 210	22.4%
	4th quintile	21 075	22.3%
	5th quintile (least deprived)	19 610	20.7%
Rural–urban classification	Urban major conurbation	14 435	15.3%
	Urban minor conurbation	6155	6.5%
	Urban city and town	49 290	52.1%
	Urban city and town in a sparse setting	235	0.3%
	Rural town and fringe	13 180	13.9%
	Rural town and fringe in a sparse setting	630	0.7%
	Rural village and dispersed	9855	10.4%
	Rural village and dispersed in a sparse setting	835	0.9%

^a^ Patient counts for subgroups were rounded to the nearest 5, so percentages were calculated as a proportion of the subgroup total.

### 3.1 Trend in DMARD monitoring rates

Throughout the study, patients missed monitoring at an average rate of 31.1% (977 354 of 3 146 849 prescribing events). The ratio of missed monitoring events to unique patients was 8.3. Between the baseline and lockdown periods, there was a significant rise in missed monitoring rates across the population, increasing from 28.4 to 40.8% (+12.4 percentage points, *P* < .001). However, between the lockdown and recovery periods, rates decreased to 28.1% (−12.7 percentage points). For a detailed breakdown of results by DMARD and monitoring test, see Table S3. For breakdowns by demographic and clinical subgroups (including statistical analysis of change over time), see Table S4.

#### 3.1.1 Medication type

The percentage of patients identified as missing monitoring was greatest for leflunomide (65.0%; 125 850 of 193 665 patients, ratio 13.5 events per unique patient) and lowest for methotrexate (24.6%; 517 770 of 2 106 050 patients, ratio 7.0 events per unique patient).

**FIGURE 1:**
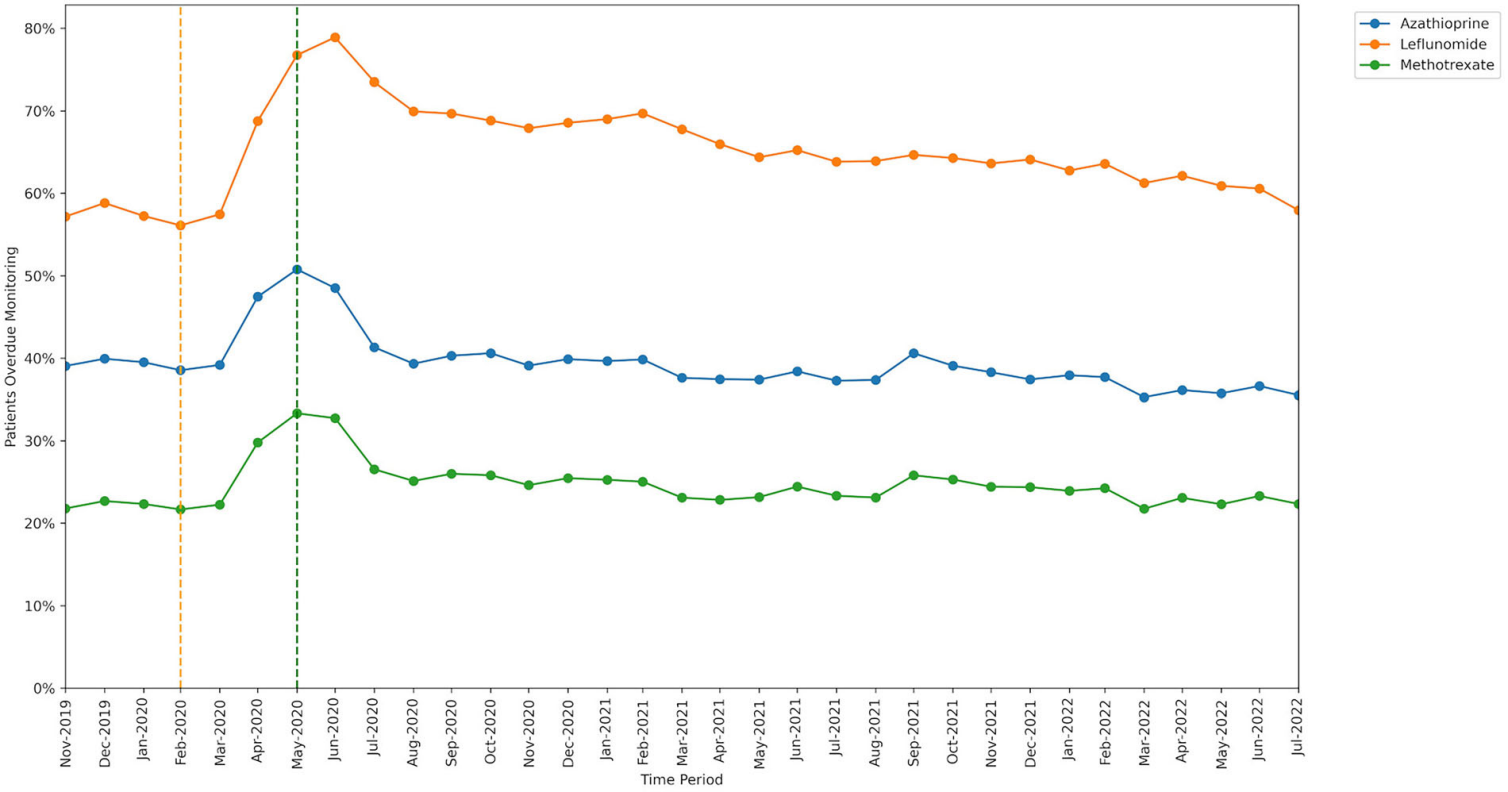
Proportion of patients overdue monitoring between November 2019 and July 2022, broken down by shared care medication. The baseline period before lockdown is shown as an orange dashed vertical line. The monitoring window, measured as 3 months from the onset of the March 2020 COVID‐19 lockdown, is shown as a green dashed vertical line.

All DMARDs exhibited an increase in missed monitoring rates immediately following the onset of lockdown (April–June 2020; Figure 1). These rates showed considerable recovery through July–August 2020. The increase in missed monitoring rates between the baseline and lockdown period was similar for methotrexate (+11.7 percentage points) and azathioprine (+12.3 percentage points), whereas a noticeably greater change was seen for leflunomide (+20.7 percentage points). By the recovery period, monitoring rates had returned to close to baseline period, in the case of azathioprine (38.5% baseline and 35.5% recovery), methotrexate (21.7% baseline and 22.3% recovery) and leflunomide (56.1% baseline and 57.9% recovery).

#### 3.1.2 Monitoring type

The monitoring test with the highest rate of missed monitoring was BP (57.4%; 111 215 of 193 665 patients, ratio 12.3 events per unique patient). All other tests had very similar rates: LFT (25.2%; 793 870 of 3 146 849 patients, ratio 7.0 events per unique patient); FBC (26.1%; 820 895 of 3 146 849 patients, ratio 7.3 events per unique patient); and U&E (25.7%; 810 255 of 3 146 849 patients, ratio 7.2 events per unique patient).

**FIGURE 2:**
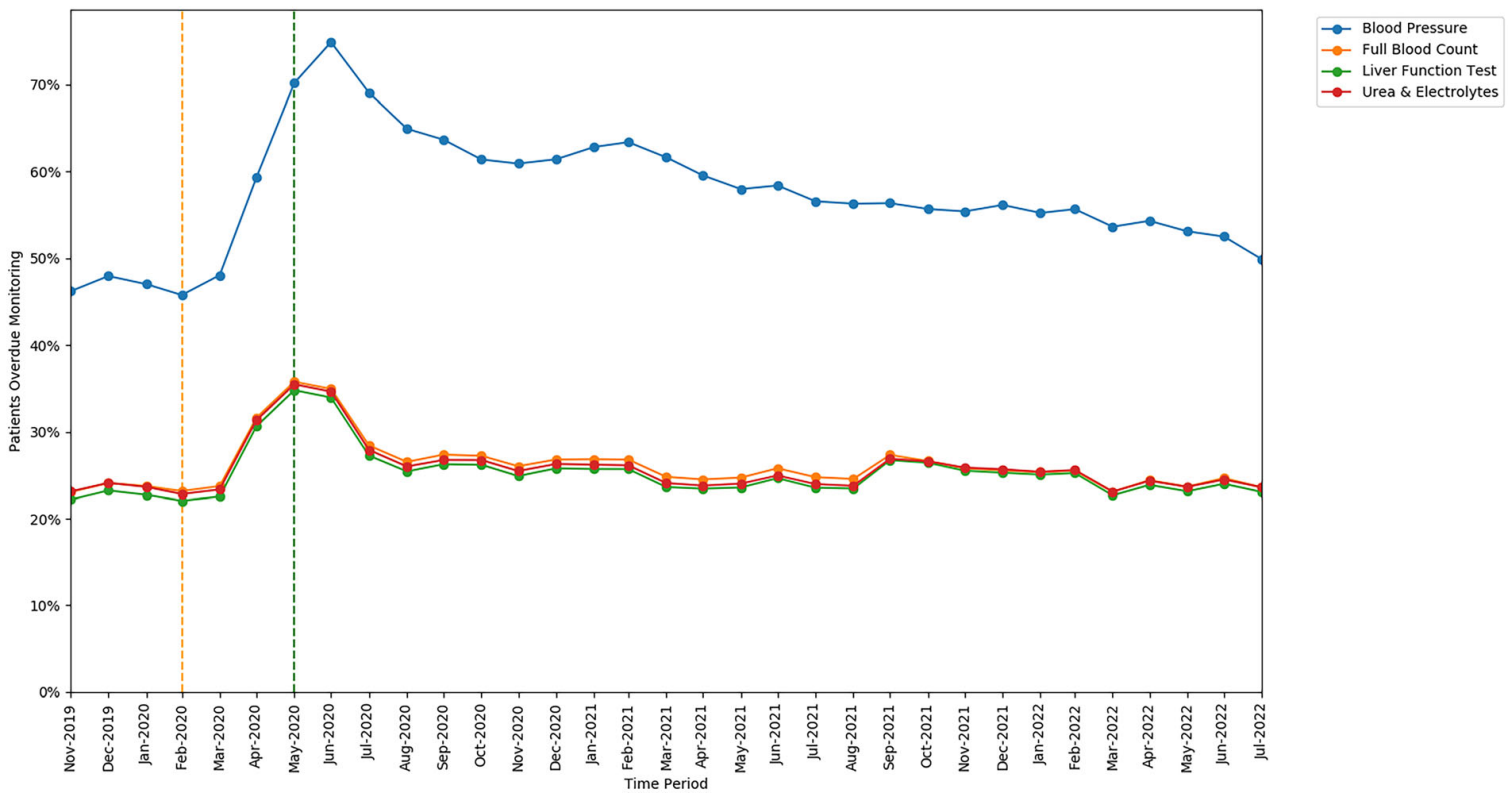
Proportions of patients overdue disease‐modifying antirheumatic drug (DMARD) monitoring between November 2019 and July 2022, broken down by monitoring test type. The baseline period before lockdown is shown as an orange dashed vertical line. The monitoring window, measured as 3 months from the onset of the March 2020 COVID‐19 lockdown, is shown as a green dashed vertical line.

All monitoring tests exhibited an increase in missed monitoring immediately following the onset of lockdown (April–June 2020; Figure 2). The increase in missed monitoring rates between the baseline and lockdown period was similar for FBC (+12.61 percentage points), LFT (+12.75 percentage points) and U&E (+12.66 percentage points), whereas a greater change was seen for BP (+24.46 percentage points). All rates showed considerable recovery through July–August 2020. By the recovery period most tests had returned to similar rates as at the baseline period: FBC (23.17% baseline and 23.58% recovery); LFT (22.04% baseline and 23.05% recovery); U&E (22.83% baseline and 23.59% recovery); with BP remaining slightly higher than baseline (45.75% baseline and 49.88% recovery).

### 3.2 Variability of change across GP practices

**FIGURE 3:**
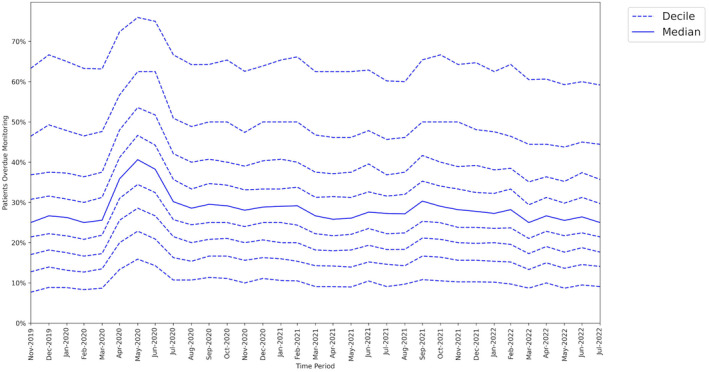
Practice level decile charts for proportions of patients overdue disease‐modifying antirheumatic drug (DMARD) monitoring between November 2019 and July 2022.

At the baseline period (February 2020), the median missed monitoring rate across GP practices was 25.0%. There was a large interdecile range, from 8.3% in the 1st decile to 63.3% in the 9th decile (Figure 3).

At the lockdown period (May 2020), the median missed monitoring rate was 40.6% (+15.6 percentage points from baseline). The interdecile range widened slightly with the 9th decile increasing by +12.6 percentage points from baseline, compared to +7.6 percentage points for the 1st decile.

At the recovery period (July 2022), the interdecile range had narrowed compared to baseline.

### 3.3 Factors associated with change in monitoring rate

Mean monitoring rates at baseline, lockdown and recovery periods are presented for each group of patient characteristics in Table S4.

**FIGURE 4:**
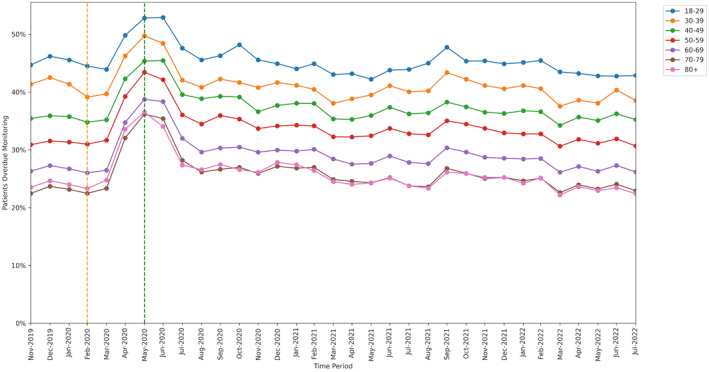
Proportions of patients overdue disease‐modifying antirheumatic drug (DMARD) monitoring between November 2019 and July 2022, broken down by age‐band. The baseline period before lockdown is shown as an orange dashed vertical line. The monitoring window, measured as 3 months from the onset of the March 2020 COVID‐19 lockdown, is shown as a green dashed vertical line.

**FIGURE 5:**
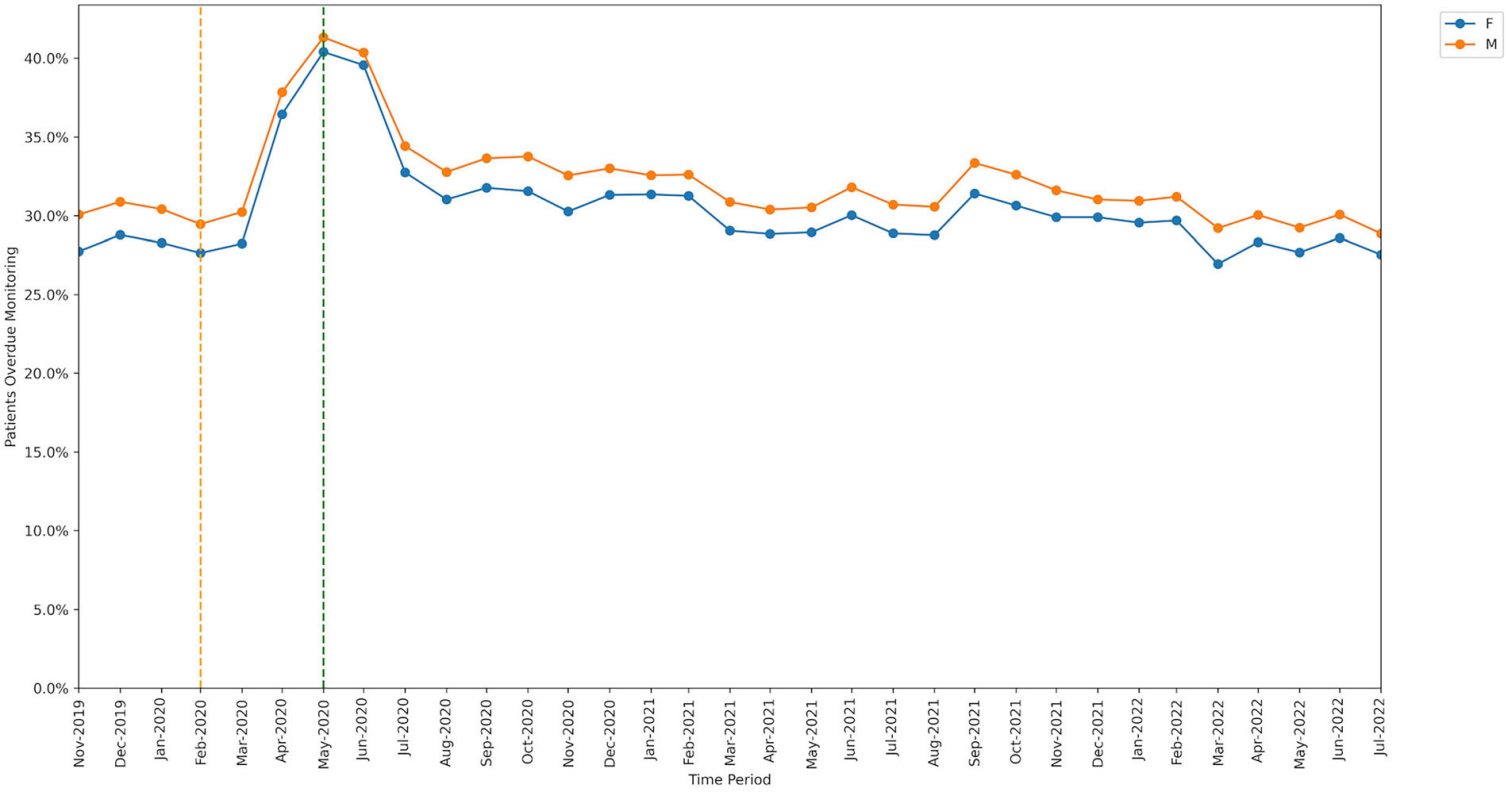
Proportions of patients overdue disease‐modifying antirheumatic drug (DMARD) monitoring between November 2019 and July 2022, broken down by sex. The baseline period before lockdown is shown as an orange dashed vertical line. The monitoring window, measured as 3 months from the onset of the March 2020 COVID‐19 lockdown, is shown as a green dashed vertical line.

**FIGURE 6:**
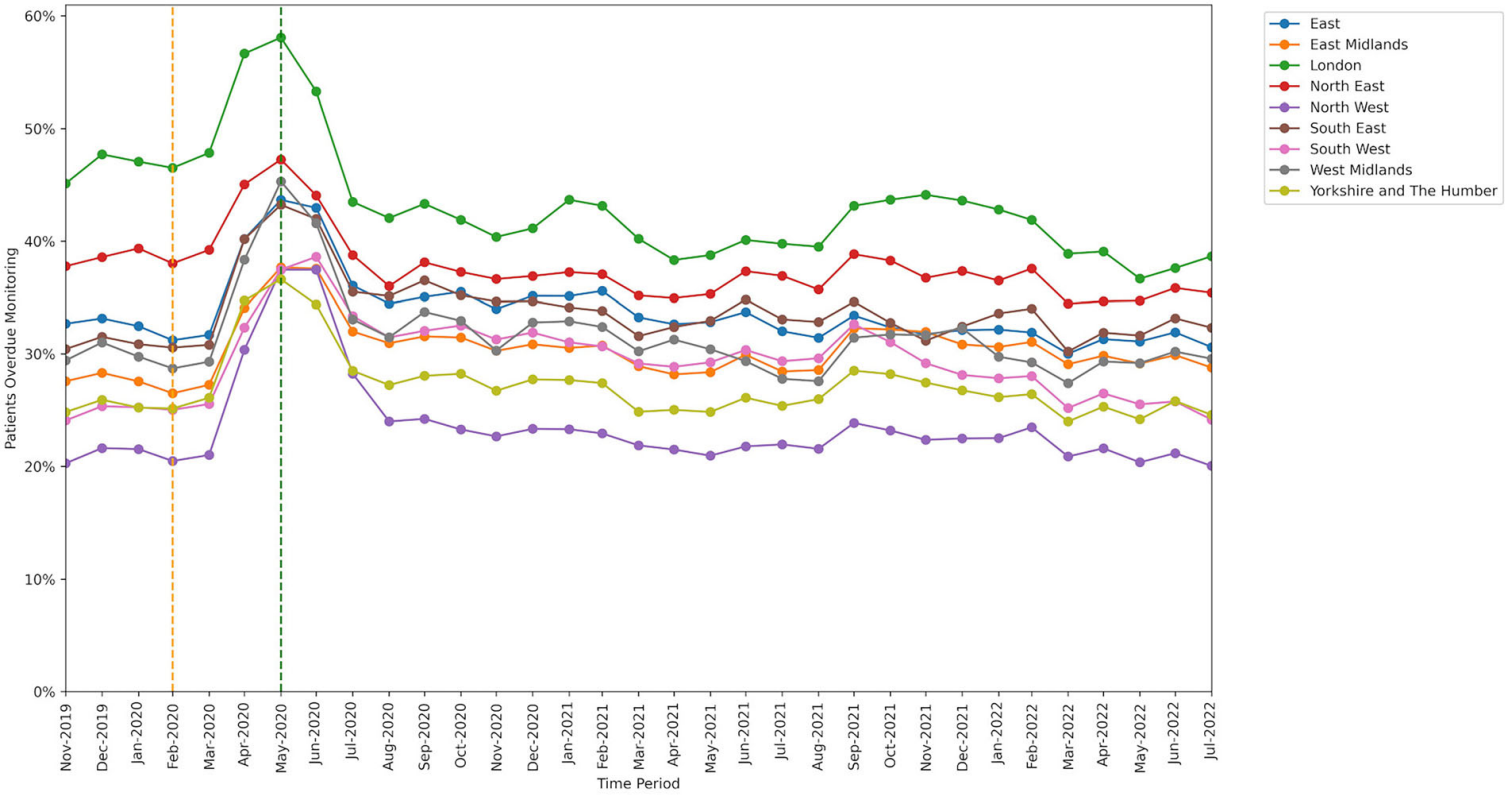
Proportions of patients overdue disease‐modifying antirheumatic drug (DMARD) monitoring between November 2019 and July 2022, broken down by region. The baseline period before lockdown is shown as an orange dashed vertical line. The monitoring window, measured as 3 months from the onset of the March 2020 COVID‐19 lockdown, is shown as a green dashed vertical line.

All groups showed significant increase in missed monitoring rates between the baseline and lockdown period. This increase differed for: age‐group categories (heterogeneity test: Cochran's Q = 37.526, *P* < .001; Figure 4), ranging from +8.3 percentage points in the 18–29 years age‐group to +13.7 percentage points in the 70–79 years age‐group; sex categories, (Q = 4.5, *P* = .034; Figure 5), ranging from +11.9 percentage points in males to +12.8 percentage points in females; and regions (Q = 78.869, *P* < .001; Figure 6), ranging from +9.2 percentage points in the North East to +17.0 percentage points in the North West.

Throughout the study, substantial overall differences were apparent in missed monitoring rates within the groups of age, sex and region (Figures 4, 5, 6) and also ethnicity, learning disabilities, serious mental illness, deprivation and rural–urban score (Figures [Link], [Link], [Link], [Link], [Link]). No substantial differences in missed monitoring rates were observed within the groups of care home, dementia or housebound statuses (Figures [Link], [Link], [Link]).

## 4 DISCUSSION

### 4.1 Summary

A significant increase in missed safety monitoring was observed following COVID‐19 lockdown measures in March 2020, suggesting that lockdowns negatively affect the ability for patients to undergo monitoring. Deterioration in monitoring was disproportionately large for: BP testing (a unique requirement for leflunomide), older people, females and specific regions. Safety monitoring recovered rapidly across all groups as lockdown measures were eased.

Throughout the study period, DMARD safety monitoring fell below the recommended standard (at least 3‐monthly checks for our selected tests), with patients being overdue for monitoring at an average rate of 31.1%. This rate was consistently higher in certain groups such as younger people, ethnic minorities, specific regions, patients living in more deprived or urban areas, and patients with an SMI or learning disability. This raises questions relating to specific health inequalities, and more generally whether current monitoring requirements are appropriate or sustainable for all patients.

### 4.2 Strengths and limitations

The scale and comprehensiveness of data in the OpenSAFELY platform is greater than any other method for accessing GP data. Historically, attempts to audit monitoring compliance in primary care would have typically relied on manual audit within a practice. By contrast, OpenSAFELY allows efficient analyses using data, which is broadly representative of the English population, providing a national overview of monitoring compliance.^22^ A second strength is the study's reproducibility and transparency. The complete set of code for the platform and all data curation/analysis from raw data to final output, is shared publicly on GitHub for peer review and efficient reuse.

We note some limitations. Firstly, our data only include results from tests carried out in primary care, or carried out in secondary care where results are returned to GPs as structured data. Test results performed outside GP practices are omitted from our data unless they are communicated to the GP practice and coded as structured data by GP practice staff. In most circumstances, where GP practices prescribe the medications, they will usually also perform the monitoring. Secondly, in some instances a clinician or the patient may have decided to stop the medication since the prescription issue date (especially during the pandemic months), which may invalidate the requirement for monitoring to be completed in the epected period. Both of these limitations may lead to some patients falsely appearing overdue for monitoring. Conversely, some tests may have been counted that were carried out for reasons unrelated to DMARD monitoring. We were also unable to capture prescriptions issued by secondary care, these data have historically been challenging to obtain.^23^ Recently, NHS Digital have made prescription data available for some hospitals—we will aim to incorporate these in future research.^24^ Lastly, we report data for 3 DMARDs, therefore findings may not be generalisable for other high‐risk drugs.

### 4.3 Comparison with existing literature

This study is the first to comprehensively evaluate DMARD monitoring at a national scale during the pandemic. Our findings are consistent with previous OpenSAFELY research, which identified deterioration in monitoring across a range of other medications and monitoring tests following the March 2020 lockdown.^13^, ^14^ For example, Fisher *et al*.^14^ reported 19.6% of patients on methotrexate missed LFT on average between January to March 2020, whilst in the current study we reported that 22.1% of patients on methotrexate missed LFT, FBC or U&E across the same period. This small difference is to be expected as our Fisher *et al*. study used indicators specific to single tests, whilst the current study included all relevant tests in a combined indicator. The difference may also be partially attributable to our study searching for a narrower range of key monitoring test codes, giving a stricter view of what is acceptable monitoring.

Research on the determinants of monitoring adherence is sparse, but parallels can be drawn with research on the determinants of medication‐taking adherence. The differences we identified in monitoring rates between certain patient groups are consistent with research suggesting that patient‐related factors (notably age, sex, ethnicity, cognitive function and socioeconomic status) can influence medication‐taking adherence.^25^ Although, it should be noted that many patient‐related factors have been reported as having an inconsistent impact, suggesting that determinants of adherence are multifaceted and context‐specific.^26^, ^27^

### 4.4 Policy implications and interpretation

This research highlights that DMARD monitoring is often not adhered to. However, wide variance in monitoring rates between practices indicates that some practices have been able to implement successful strategies. This presents opportunities for sharing best practice, which NHS commissioners may be able to facilitate at scale.

The deterioration that this study observed in DMARD monitoring during lockdown, and that other studies have observed in routine services,^13^ offers insight for health policymakers regarding the effects of the pandemic and its management strategies. A combination of factors probably contributed to this deterioration including: diversion of primary care resources towards managing COVID‐19, guidance from national bodies to enable reduced monitoring frequency, supply problems with blood sample bottles and patients choosing to avoid healthcare for fear of exposure to COVID‐19. This understanding will help inform assessment of the risks and benefits of lockdown measures in future pandemics, and how the impact on groups that were more severely affected could be mitigated, through strategies that preserve patient engagement and accessibility.

The disproportionately large deterioration observed in BP monitoring may suggest that clinicians deemed it less critical than blood tests, reflecting appropriate prioritization of monitoring. Regional differences in deterioration may suggest that regions prioritized DMARD monitoring differently during the pandemic. Factors underpinning regional differences may include the availability of local workforce and proximity of facilities to support the pandemic response; both have been suggested as relevant to service resilience during the pandemic.^28^ Differences in resilience may also be related to known variability in the robustness of local shared care processes and documentation,^29^ and regional differences which have been observed in the time between first rheumatoid arthritis review to DMARD initiation.^30^

The overall differences in monitoring rates observed between various groups highlight ongoing inequalities. For example, younger age‐groups generally missed monitoring more often than older age‐groups. This may indicate that younger age‐groups exhibit decreased engagement with monitoring, which is consistent with previous research.^31^ Less monitoring in younger‐age groups means there are fewer opportunities for corrective dose adjustments, which may help explain findings from a recent study: that younger patients taking methotrexate or azathioprine were more likely to have abnormal monitoring test results when tested.^32^ However, it should be noted that older age‐groups are more vulnerable to DMARD toxicity.^33^

Health commissioners should consider targeted strategies to reduce inequalities as appropriate, for example, addressing the disproportionately high rate of missed BP monitoring (often recurring in the same patients on leflunomide) may be a pragmatic starting point. Since the pandemic, standardized national processes for shared care have been published by NHS England in relation to several medicines,^34^ adopting these national standards should help local health commissioners reduce inequalities.

This analysis has substantial implications for NHS data infrastructure. Historically, practical and privacy challenges around accessing primary care data meant that national audits relied upon manual data collection by local teams and central collation—an approach with high resource and time costs. The OpenSAFELY platform enabled us to execute a single analysis for a representative sample of the population in near‐real‐time (with data available in OpenSAFELY only 2–9 days after being entered by a clinician) whilst leaving data in situ. This efficient approach allows our analysis to be easily extended (by building upon open‐access code) to wider populations, or to provide more granular data on demographic or clinical subgroups. Ultimately, OpenSAFELY provides tools which facilitate rapid detailed feedback to NHS commissioners and clinicians, enabling timely and meaningful interventions to improve patient outcomes.

### 4.5 Future research

To inform policy interventions, further research is needed to explore the causes of poorer performance amongst specific practices and patient groups. This could be done through qualitative studies surveying/interviewing those with poorer monitoring rates to identify the individual traits, attitudes, behaviours and circumstances underpinning their poorer monitoring adherence.

More broadly, the pandemic offers an unprecedented opportunity of a natural experiment, since changes to clinical practice (such as the frequency of safety monitoring) have occurred that would have been unethical in normal circumstances. This allows researchers to test whether certain principles of clinical practice could be optimized. Recent research has suggested that the clinical impact of missing DMARD monitoring is minimal, with 1 study suggesting that reducing the frequency of methotrexate monitoring from 3 to 6 monthly did not increase abnormal test results nor cause harm to patients.^35^ However, such analysis is easily confounded by patient‐specific factors related to clinical risk, which influence the likelihood of monitoring occurring. For example, patients at lower risk of negative outcomes may be less motivated to attend monitoring, or less proactively followed up by clinicians. By contrast, patients at higher risk may be more likely to have accessibility issues inhibiting monitoring, or more reluctant to attend monitoring due to fear of contracting COVID‐19 with their clinical vulnerability. Consequently, patients who miss monitoring may have an intrinsically different likelihood of negative outcomes. Studies could attempt to evaluate the clinical impact of reduced monitoring frequency within patient groups stratified by clinical risk factors, and break results down for various medications/tests. This may reveal whether certain medications or patient groups benefit from monitoring to different extents, informing more tailored cost‐effective strategies for who should receive which monitoring tests, and how often.

### 4.6 Conclusion

A transient deterioration in DMARD safety monitoring occurred across all groups during the COVID‐19 pandemic, acutely around the time of lockdown measures with performance mostly recovering rapidly as lockdown measures were eased. Long‐term differences in safety monitoring rates exist between several sociodemographic and clinical subgroups, and the pandemic impacted monitoring in these populations to varying extents. Health commissioners should identify causes of poorer monitoring in specific groups and consider implementing supportive measures. Monitoring rates varied substantially between practices, suggesting opportunities exist to improve service consistency by sharing good practice and adopting standardized shared care processes. These analyses demonstrate the capability of the OpenSAFELY platform as an effective tool to provide actionable insights on health service provision and inequalities.

## AUTHOR CONTRIBUTIONS

*Conceptualization*: Andrew D. Brown, Ruth E. Costello and Brian MacKenna. *Data curation*: Andrew D. Brown, Louis Fisher, Lisa E.M. Hopcroft, Christine Cunningham, Seb C.J. Bacon, Amir Mehrkar, George Hickman, Chris Bates, Jonathan Cockburn, John Parry, Frank Hester and Sam Harper. *Formal analysis*: Andrew D. Brown, Louis Fisher, Milan Wiedemann, William J. Hulme and Helen J. Curtis. *Funding acquisition*: Ben Goldacre. *Investigation*: Andrew D. Brown, Louis Fisher, Milan Wiedemann, William J. Hulme and Helen J. Curtis. *Methodology*: Andrew D. Brown, William J. Hulme and Brian MacKenna. *Project administration*: Amir Mehrkar. *Resources*: Amir Mehrkar, Seb C.J. Bacon, George Hickman, Chris Bates, Jonathan Cockburn, John Parry, Frank Hester and Sam Harper. *Software*: Seb C.J. Bacon, George Hickman, Chris Bates, Jonathan Cockburn, John Parry, Frank Hester and Sam Harper. *Supervision*: Zeyneb Kurt, Amir Mehrkar, Ben Goldacre and Brian MacKenna. *Visualization*: Andrew D. Brown, Louis Fisher and Brian MacKenna. *Writing—original draft*: Andrew D. Brown. *Writing—review and editing*: Andrew D. Brown, Louis Fisher, Helen J. Curtis, Milan Wiedemann, William J. Hulme, Lisa E.M. Hopcroft, Christine Cunningham, Victoria Speed, Ruth E. Costello, James B. Galloway, Mark D. Russell, Katie Bechman, Zeyneb Kurt, Richard Croker, Chris Wood, Alex J. Walker, Andrea L. Schaffer, Seb C.J. Bacon, Amir Mehrkar, George Hickman, Ben Goldacre and Brian MacKenna.

## CONFLICT OF INTEREST STATEMENT

All authors have completed the ICMJE uniform disclosure form at www.icmje.org/coi_disclosure.pdf. B.G. has received research funding from the Laura and John Arnold Foundation, the NHS National Institute for Health Research (NIHR), the NIHR School of Primary Care Research, the NIHR Oxford Biomedical Research Centre, the Mohn‐Westlake Foundation, NIHR Applied Research Collaboration Oxford and Thames Valley, the Wellcome Trust, the Good Thinking Foundation, Health Data Research UK, the Health Foundation, the World Health Organisation, UKRI, Asthma UK, the British Lung Foundation, and the Longitudinal Health and Wellbeing strand of the National Core Studies programme; he also receives personal income from speaking and writing for lay audiences on the misuse of science. B.M.K. is employed by NHS England working on medicines policy and clinical lead for primary care medicines data. C.B., J.C., J.P., F.H. and S.H. are employees of TPP.

## INFORMATION GOVERNANCE

NHS England is the data controller; TPP is the data processor; and the researchers on OpenSAFELY are acting with the approval of NHS England. This implementation of OpenSAFELY is hosted within the TPP environment which is accredited to the ISO 27001 information security standard and is NHS IG Toolkit compliant;^36^, ^37^ patient data have been pseudonymized for analysis and linkage using industry standard cryptographic hashing techniques; all pseudonymized datasets transmitted for linkage onto OpenSAFELY are encrypted; access to the platform is via a virtual private network connection, restricted to a small group of researchers; the researchers hold contracts with NHS England and only access the platform to initiate database queries and statistical models; all database activity is logged; only aggregate statistical outputs leave the platform environment following best practice for anonymization of results such as statistical disclosure control for low cell counts.^38^ The OpenSAFELY research platform adheres to the obligations of the UK General Data Protection Regulation (GDPR) and the Data Protection Act 2018. In March 2020, the Secretary of State for Health and Social Care used powers under the UK Health Service (Control of Patient Information) Regulations 2002 (COPI) to require organizations to process confidential patient information for the purposes of protecting public health, providing healthcare services to the public and monitoring and managing the COVID‐19 outbreak and incidents of exposure; this sets aside the requirement for patient consent.^39^ Taken together, these provide the legal bases to link patient datasets on the OpenSAFELY platform. GP practices, from which the primary care data are obtained, are required to share relevant health information to support the public health response to the pandemic, and have been informed of the OpenSAFELY analytics platform.

## Supporting information


**FIGURE S1.** Proportions of patients overdue DMARD monitoring between November 2019 and July 2022, broken down by ethnicity. The baseline period before lockdown is shown as an orange dashed vertical line. The monitoring window, measured as 3 months from the onset of the March 2020 COVID‐19 lockdown, is shown as a green dashed vertical line.

**FIGURE S2.** Proportions of patients overdue DMARD monitoring between November 2019 and July 2022, broken down by whether a learning disability diagnosis was coded. The baseline period before lockdown is shown as an orange dashed vertical line. The monitoring window, measured as 3 months from the onset of the March 2020 COVID‐19 lockdown, is shown as a green dashed vertical line.

**FIGURE S3.** Proportions of patients overdue DMARD monitoring between November 2019 and July 2022, broken down by whether a serious mental illness diagnosis was coded. The baseline period before lockdown is shown as an orange dashed vertical line. The monitoring window, measured as 3 months from the onset of the March 2020 COVID‐19 lockdown, is shown as a green dashed vertical line.

**FIGURE S4.** Proportions of patients overdue DMARD monitoring between November 2019 and July 2022, broken down by Index of Multiple Deprivation quintile. The baseline period before lockdown is shown as an orange dashed vertical line. The monitoring window, measured as 3 months from the onset of the March 2020 COVID‐19 lockdown, is shown as a green dashed vertical line.

**FIGURE S5.** Proportions of patients overdue DMARD monitoring between November 2019 and July 2022, broken down by rural–urban score band. The baseline period before lockdown is shown as an orange dashed vertical line. The monitoring window, measured as 3 months from the onset of the March 2020 COVID‐19 lockdown, is shown as a green dashed vertical line.

**FIGURE S6.** Proportions of patients overdue DMARD monitoring between November 2019 and July 2022, broken down by whether patients were coded as residing in a care home. The baseline period before lockdown is shown as an orange dashed vertical line. The monitoring window, measured as 3 months from the onset of the March 2020 COVID‐19 lockdown, is shown as a green dashed vertical line.

**FIGURE S7.** Proportions of patients overdue DMARD monitoring between November 2019 and July 2022, broken down by whether a dementia diagnosis was coded. The baseline period before lockdown is shown as an orange dashed vertical line. The monitoring window, measured as 3 months from the onset of the March 2020 COVID‐19 lockdown, is shown as a green dashed vertical line.

**FIGURE S8.** Proportions of patients overdue DMARD monitoring between November 2019 and July 2022, broken down by whether patients were coded as housebound. The baseline period before lockdown is shown as an orange dashed vertical line. The monitoring window, measured as 3 months from the onset of the March 2020 COVID‐19 lockdown, is shown as a green dashed vertical line.

**TABLE S1.** Key features which were assessed to determine the completion of monitoring tests, and links to codelists which identified these features.
**TABLE S2.** Indicator definitions.
**TABLE S3.** DMARD missed monitoring rates at baseline (Dec19 to Feb20), lockdown (Mar20 to May20) and recovery (May22 to Jul22), with cumulative counts of missed monitoring events and unique patients experiencing a missed monitoring event over the full study period (Nov19 to Jul22), broken down by medication and monitoring test.
**TABLE S4.** DMARD missed monitoring rates* for the population broken down into demographic and clinical characteristics at baseline (Dec19 to Feb20), lockdown (Mar20 to May20) and recovery (May22 to Jul22).

## Data Availability

Access to the underlying identifiable and potentially re‐identifiable pseudonymized electronic health record data is tightly governed by various legislative and regulatory frameworks, and restricted by best practice. The data in OpenSAFELY is drawn from General Practice data across England where TPP is the Data Processor. TPP developers initiate an automated process to create pseudonymized records in the core OpenSAFELY database, which are copies of key structured data tables in the identifiable records. These pseudonymized records are linked onto key external data resources that have also been pseudonymized via SHA‐512 1‐way hashing of NHS numbers using a shared salt. Bennett Institute for Applied Data Science developers and PIs holding contracts with NHS England have access to the OpenSAFELY pseudonymized data tables as needed to develop the OpenSAFELY tools. These tools in turn enable researchers with OpenSAFELY Data Access Agreements to write and execute code for data management and data analysis without direct access to the underlying raw pseudonymized patient data, and to review the outputs of this code. All code for the full data management pipeline—from raw data to completed results for this analysis—and for the OpenSAFELY platform as a whole is available for review at github.com/OpenSAFELY.
