# Supplementary figures and images for: OpenSAFELY: The impact of COVID‐19 on azathioprine, leflunomide and methotrexate monitoring, and factors associated with change in monitoring rate
Source: Br J Clin Pharmacol. 2024 Apr 8;91(6):1586–99. doi: 10.1111/bcp.16062 (PMC7616619; doi:10.1111/bcp.16062)

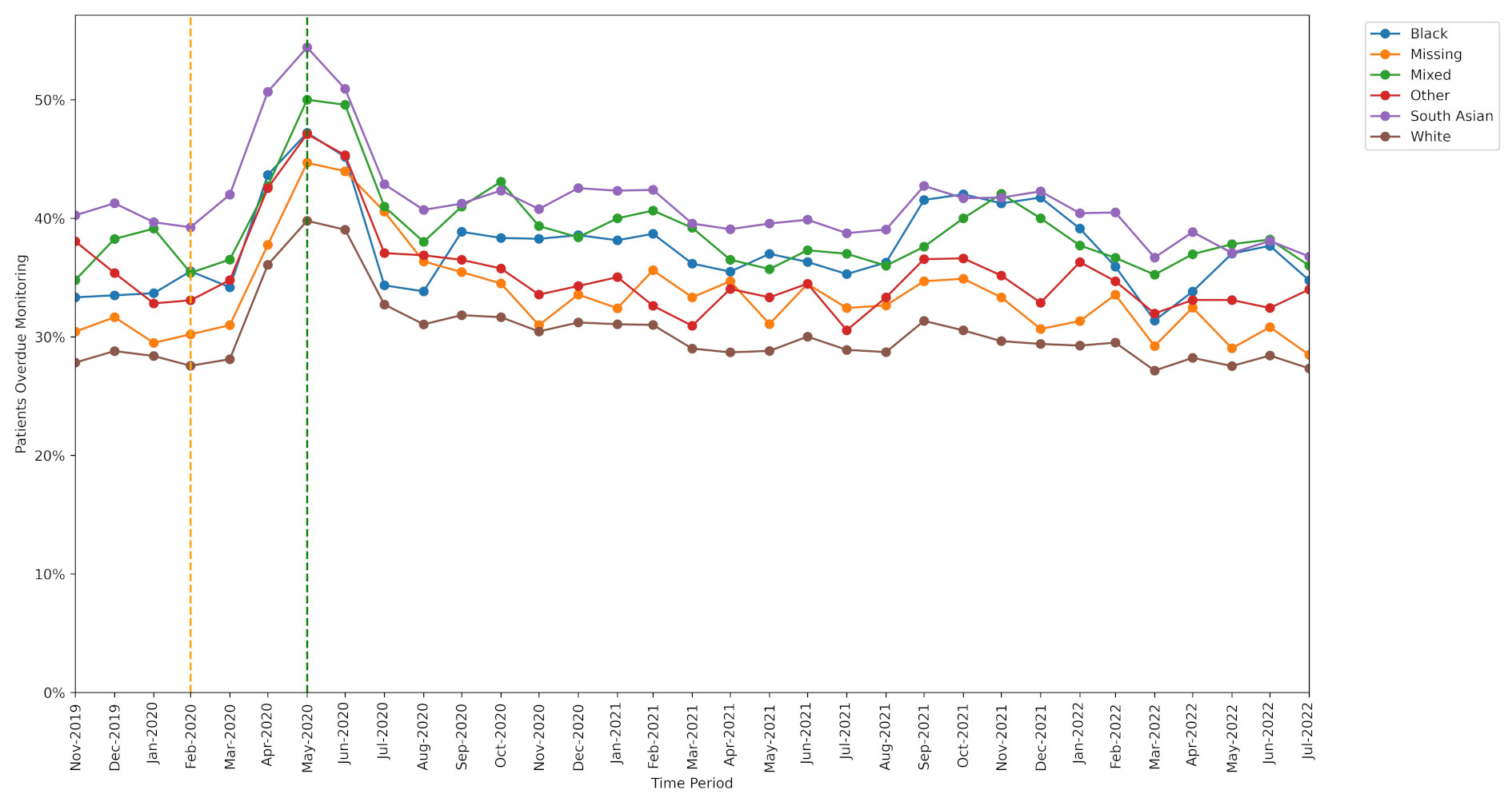

Supplement: Supplementary file 1 — FIGURE S1. Proportions of patients overdue DMARD monitoring between November 2019 and July 2022, broken down by ethnicity. The baseline period before lockdown is shown as an orange dashed vertical line. The monitoring window, measured as 3 months from the onset of the March 2020 COVID‐19 lockdown, is shown as a green dashed vertical line. [file BCP-91-1586-s009.jpeg]

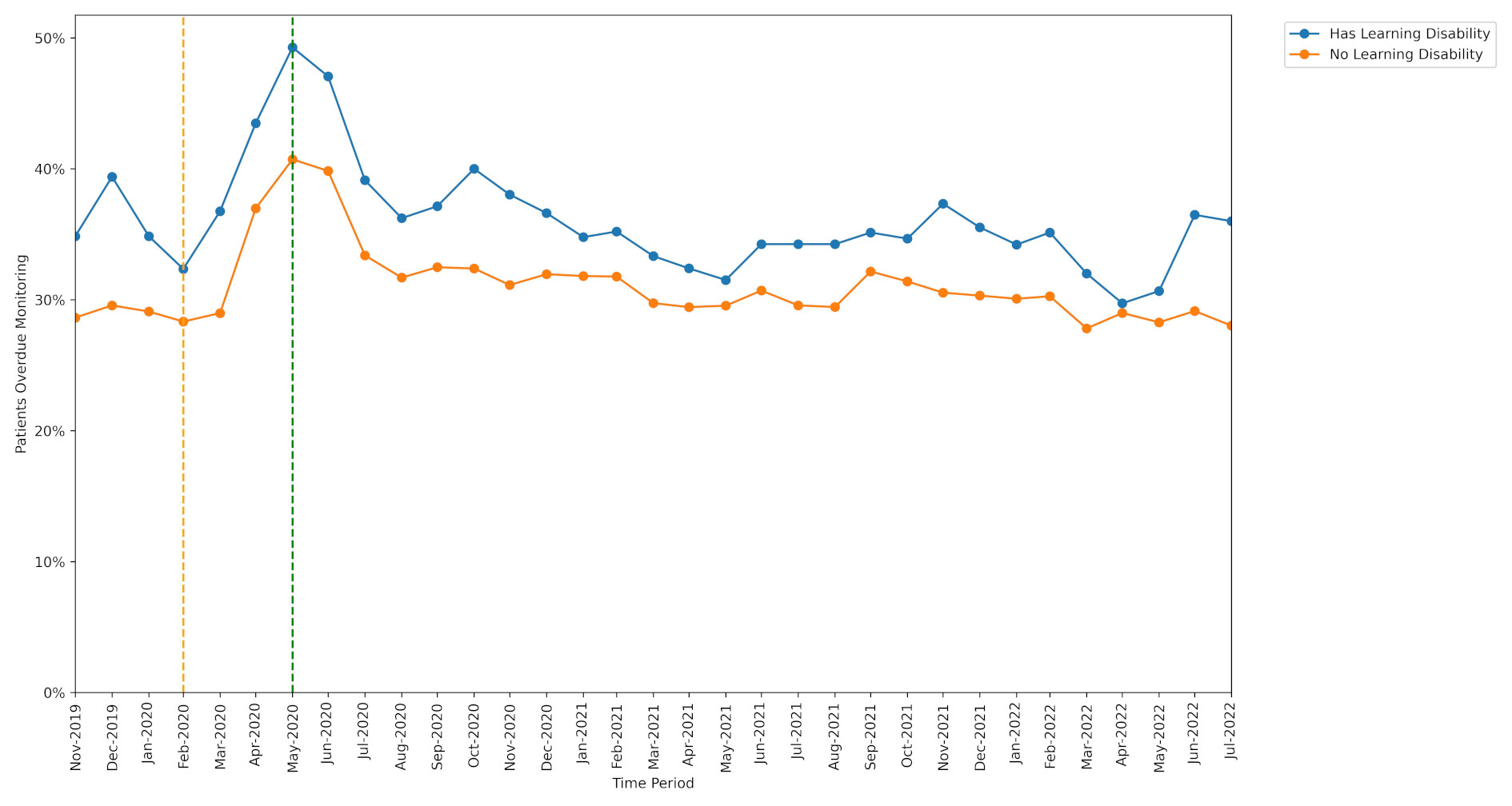

Supplement: Supplementary file 2 — FIGURE S2. Proportions of patients overdue DMARD monitoring between November 2019 and July 2022, broken down by whether a learning disability diagnosis was coded. The baseline period before lockdown is shown as an orange dashed vertical line. The monitoring window, measured as 3 months from the onset of the March 2020 COVID‐19 lockdown, is shown as a green dashed vertical line. [file BCP-91-1586-s004.jpeg]

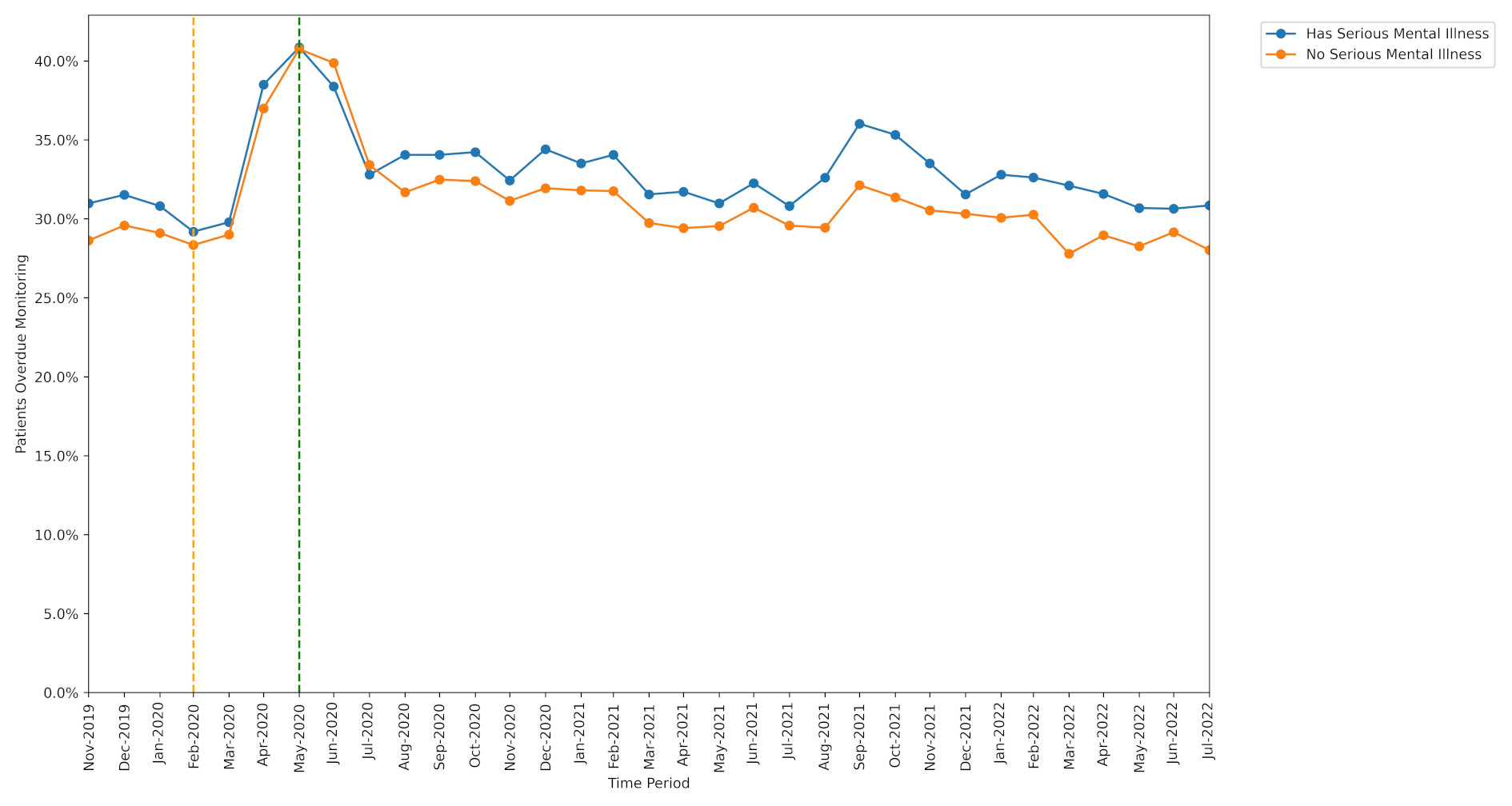

Supplement: Supplementary file 3 — FIGURE S3. Proportions of patients overdue DMARD monitoring between November 2019 and July 2022, broken down by whether a serious mental illness diagnosis was coded. The baseline period before lockdown is shown as an orange dashed vertical line. The monitoring window, measured as 3 months from the onset of the March 2020 COVID‐19 lockdown, is shown as a green dashed vertical line. [file BCP-91-1586-s008.jpeg]

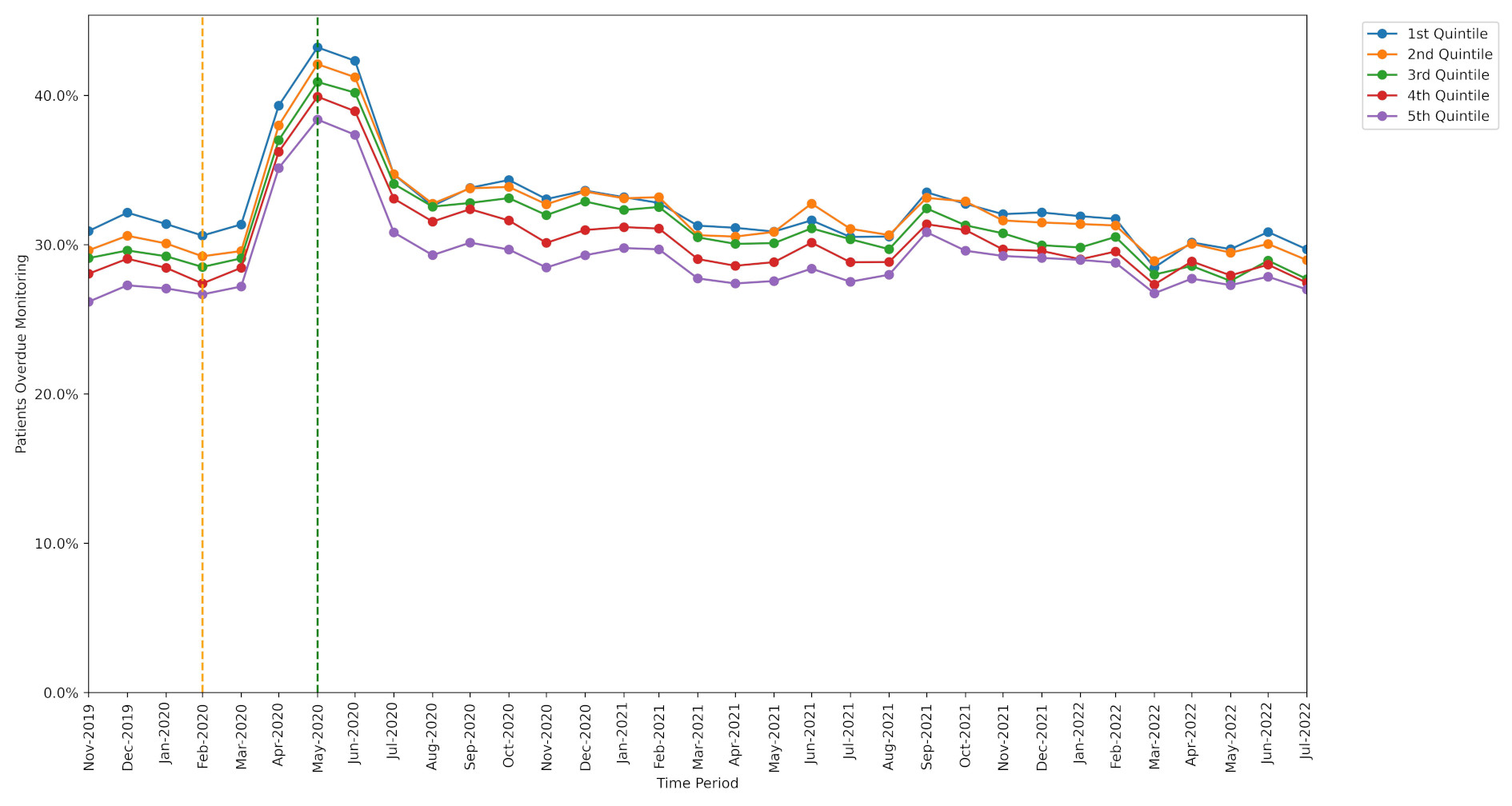

Supplement: Supplementary file 4 — FIGURE S4. Proportions of patients overdue DMARD monitoring between November 2019 and July 2022, broken down by Index of Multiple Deprivation quintile. The baseline period before lockdown is shown as an orange dashed vertical line. The monitoring window, measured as 3 months from the onset of the March 2020 COVID‐19 lockdown, is shown as a green dashed vertical line. [file BCP-91-1586-s002.jpeg]

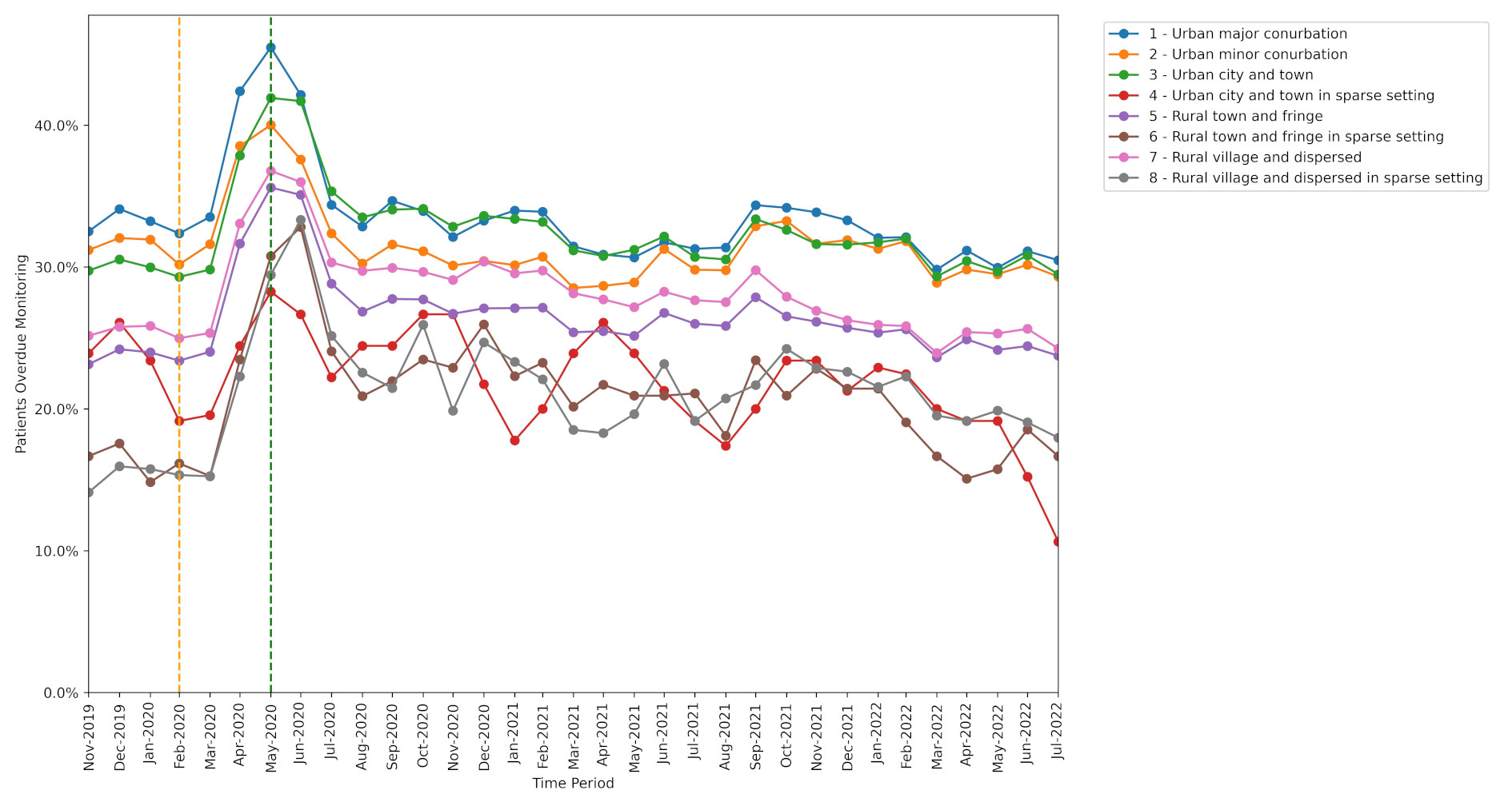

Supplement: Supplementary file 5 — FIGURE S5. Proportions of patients overdue DMARD monitoring between November 2019 and July 2022, broken down by rural–urban score band. The baseline period before lockdown is shown as an orange dashed vertical line. The monitoring window, measured as 3 months from the onset of the March 2020 COVID‐19 lockdown, is shown as a green dashed vertical line. [file BCP-91-1586-s006.jpeg]

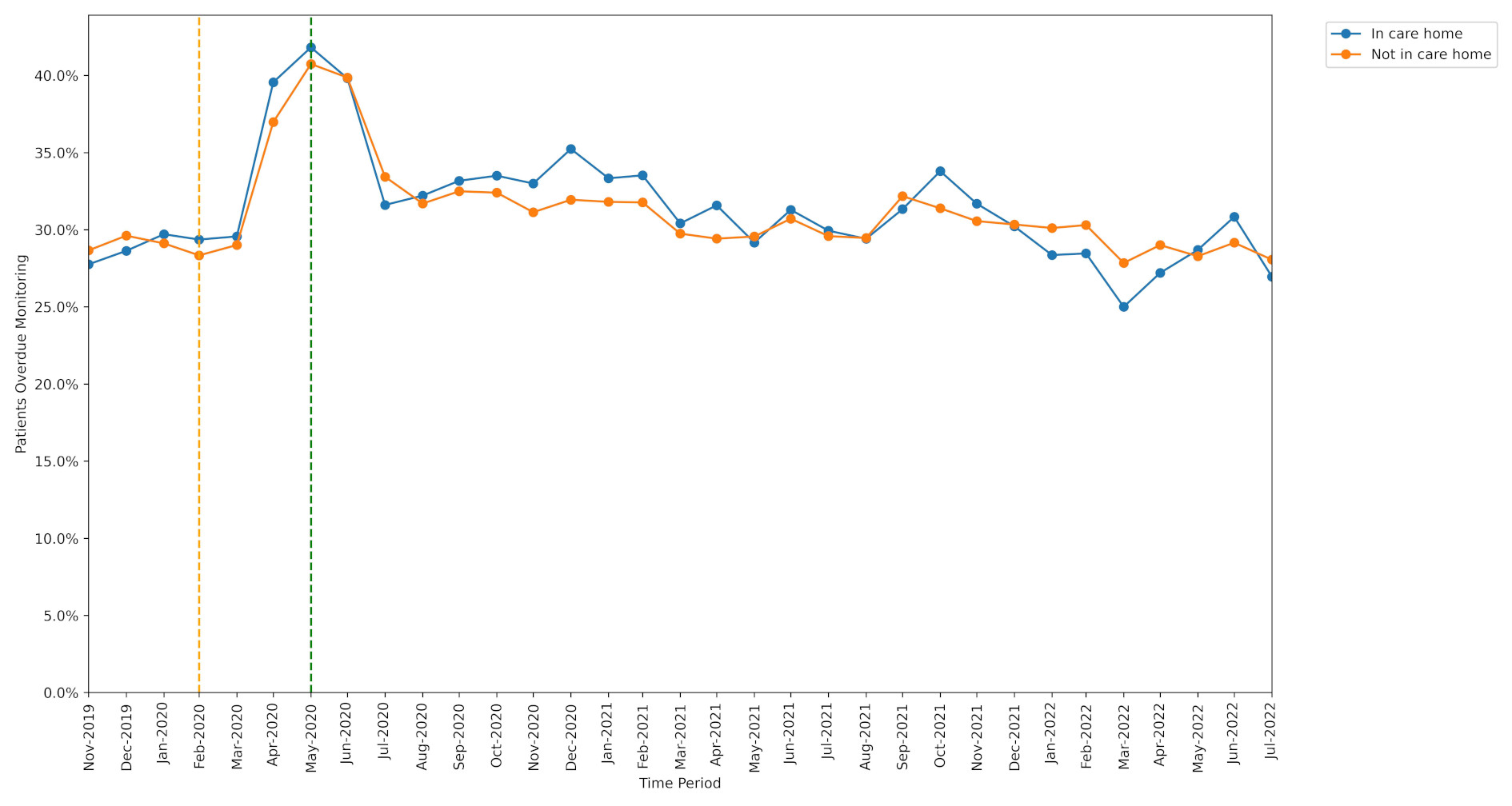

Supplement: Supplementary file 6 — FIGURE S6. Proportions of patients overdue DMARD monitoring between November 2019 and July 2022, broken down by whether patients were coded as residing in a care home. The baseline period before lockdown is shown as an orange dashed vertical line. The monitoring window, measured as 3 months from the onset of the March 2020 COVID‐19 lockdown, is shown as a green dashed vertical line. [file BCP-91-1586-s003.jpeg]

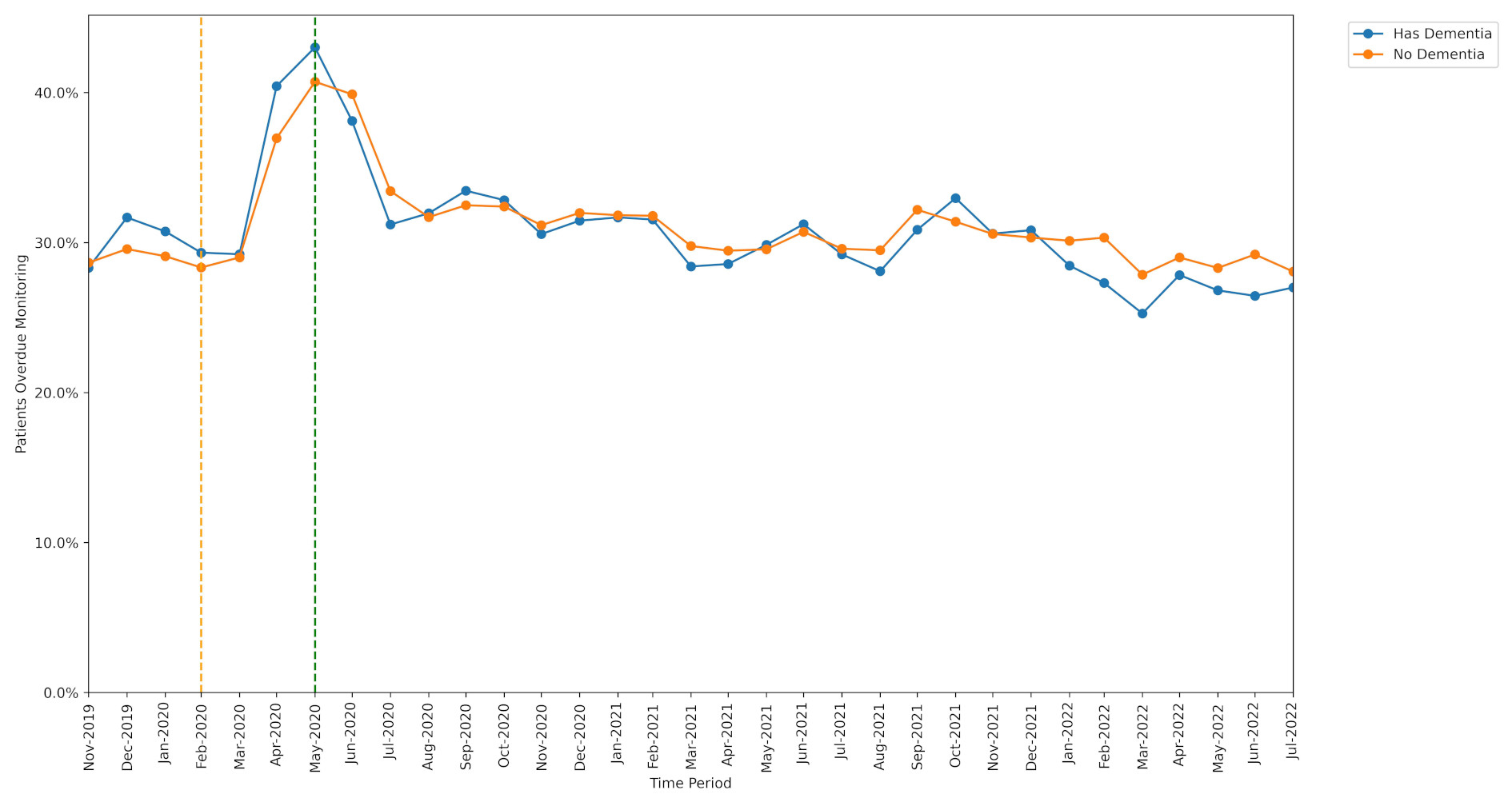

Supplement: Supplementary file 7 — FIGURE S7. Proportions of patients overdue DMARD monitoring between November 2019 and July 2022, broken down by whether a dementia diagnosis was coded. The baseline period before lockdown is shown as an orange dashed vertical line. The monitoring window, measured as 3 months from the onset of the March 2020 COVID‐19 lockdown, is shown as a green dashed vertical line. [file BCP-91-1586-s001.jpeg]

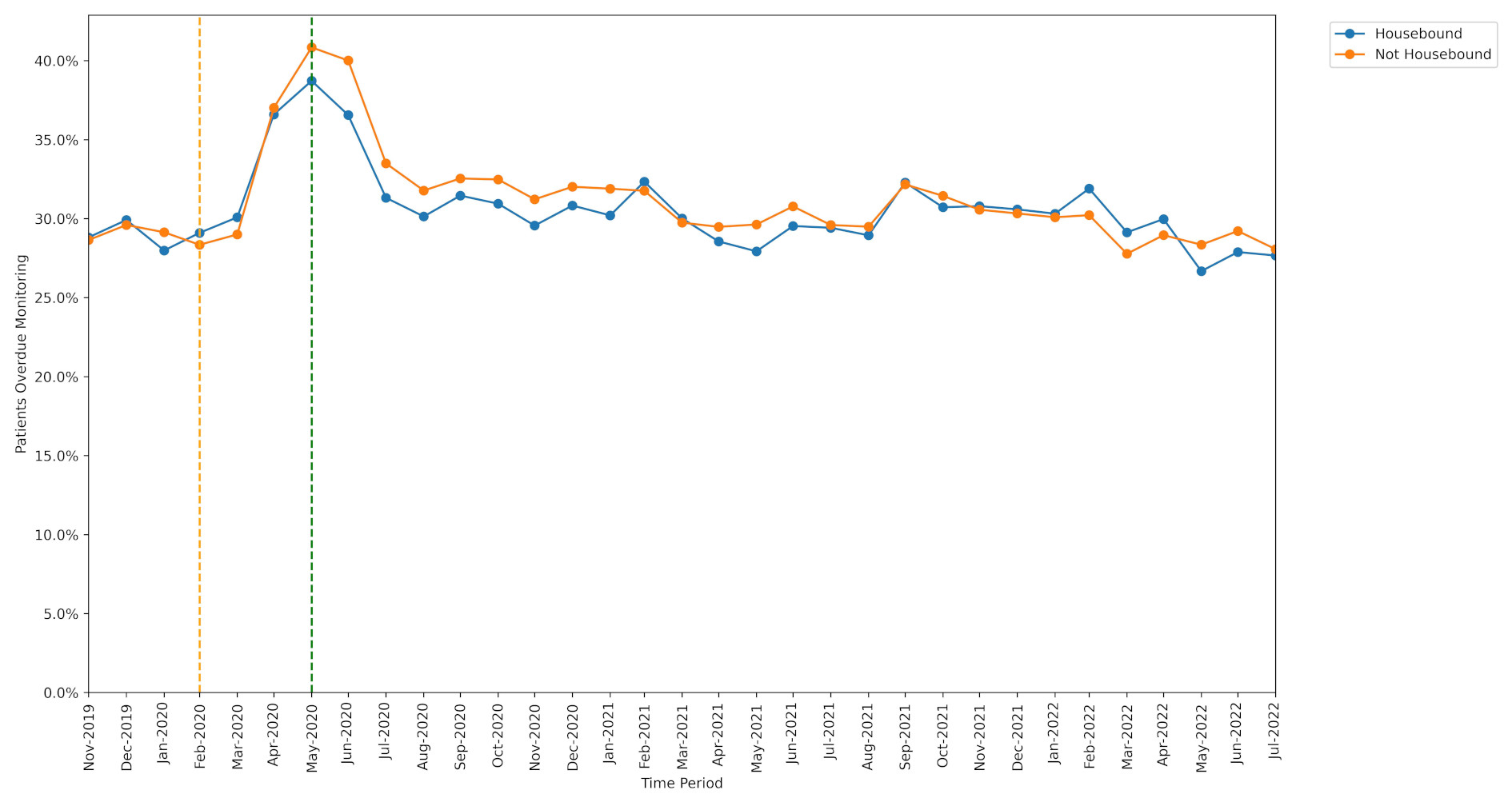

Supplement: Supplementary file 8 — FIGURE S8. Proportions of patients overdue DMARD monitoring between November 2019 and July 2022, broken down by whether patients were coded as housebound. The baseline period before lockdown is shown as an orange dashed vertical line. The monitoring window, measured as 3 months from the onset of the March 2020 COVID‐19 lockdown, is shown as a green dashed vertical line. [file BCP-91-1586-s007.jpeg]
