# Supplementary material for: OpenSAFELY: The impact of COVID‐19 on azathioprine, leflunomide and methotrexate monitoring, and factors associated with change in monitoring rate
Source: Br J Clin Pharmacol. 2024 Apr 8;91(6):1586–99. doi: 10.1111/bcp.16062 (PMC7616619; doi:10.1111/bcp.16062)
Supplement: Supplementary file 9 — TABLE S1. Key features which were assessed to determine the completion of monitoring tests, and links to codelists which identified these features. TABLE S2. Indicator definitions. TABLE S3. DMARD missed monitoring rates at baseline (Dec19 to Feb20), lockdown (Mar20 to May20) and recovery (May22 to Jul22), with cumulative counts of missed monitoring events and unique patients experiencing a missed monitoring event over the full study period (Nov19 to Jul22), broken down by medication and monitoring test. TABLE S4. DMARD missed monitoring rates* for the population broken down into demographic and clinical characteristics at baseline (Dec19 to Feb20), lockdown (Mar20 to May20) and recovery (May22 to Jul22). [file BCP-91-1586-s005.docx]

Table S1. Key features which were assessed to determine the completion of monitoring tests, and links to codelists which identified these features.

| **Monitoring Test** | **Representative Feature** | **Codelist** |
| --- | --- | --- |
| Urea and electrolytes (U&E) | Sodium level | <https://www.opencodelists.org/codelist/opensafely/sodium-tests/254b9f34/> |
| Full blood count (FBC) | Red blood cell count | <https://www.opencodelists.org/codelist/opensafely/red-blood-cell-rbc-tests/576a859e/> |
| Liver function test (LFT) | Alanine-aminotransferase level | <https://www.opencodelists.org/codelist/opensafely/alanine-aminotransferase-alt-tests/2298df3e/> |
| Blood pressure (BP) | Systolic blood pressure | <https://www.opencodelists.org/codelist/opensafely/systolic-blood-pressure-qof/3572b5fb/> |

Table S2. Indicator definitions.

| **Research Question** | **Description** | **Denominator** | **Numerator** |
| --- | --- | --- | --- |
| 1.1 Did the monitoring rate for DMARDs change during the pandemic? | **DMARD Missed Monitoring Rate:** Proportion of patients taking Methotrexate, Leflunomide or Azathioprine who were overdue at least one monitoring test. | Patients who had a prescription issued for Methotrexate, Leflunomide or Azathioprine in the time-periods^*^ of interest. | Patients in the denominator who did not have a value recorded for all the following in the 3 months prior to the search index date: FBC, LFT, U&E's, Blood Pressure (for Leflunomide only). |
| 1.2 Did specific medications miss monitoring more often than others during the pandemic? | **Methotrexate Missed Monitoring Rate:** Proportion of patients taking Methotrexate who were overdue at least one monitoring test. | Patients who had a prescription issued for Methotrexate in the time-periods^*^ of interest. | Patients in the denominator who did not have a value recorded for all the following in the 3 months prior to the search index date: FBC, LFT, U&E's. |
|  | **Leflunomide Missed Monitoring Rate:** Proportion of patients taking Leflunomide who were overdue at least one monitoring test. | Patients who had a prescription issued for Leflunomide in the time-periods^*^ of interest. | Patients in the denominator who did not have a value recorded for all the following in the 3 months prior to the search index date: FBC, LFT, U&E's, Blood Pressure. |
|  | **Azathioprine Missed Monitoring Rate:** Proportion of patients taking Azathioprine who were overdue at least one monitoring test. | Patients who had a prescription issued for Azathioprine in the time-periods^*^ of interest. | Patients in the denominator who did not have a value recorded for all the following in the 3 months prior to the search index date: FBC, LFT, U&E's. |
| 1.3 Were specific tests missed more often than others during the pandemic? | **FBC Missed Monitoring Rate:** Proportion of required full blood count tests that were not completed. | Patients who had a prescription issued for Methotrexate, Leflunomide or Azathioprine in the time-periods^*^ of interest. | Patients in the denominator who did not have a value recorded for FBC in the 3 months prior to the search index date. |
|  | **LFT Missed Monitoring Rate:** Proportion of required liver function tests that were not completed. | Patients who had a prescription issued for Methotrexate, Leflunomide or Azathioprine in the time-periods^*^ of interest. | Patients in the denominator who did not have a value recorded for LFT in the 3 months prior to the search index date. |
|  | **U&E Missed Monitoring Rate:** Proportion of required urea and electrolyte tests that were not completed. | Patients who had a prescription issued for Methotrexate, Leflunomide or Azathioprine in the time-periods^*^ of interest. | Patients in the denominator who did not have a value recorded for U&E in the 3 months prior to the search index date. |
|  | **BP Missed Monitoring Rate:** Proportion of required blood pressure tests that were not completed. | Patients who had a prescription issued for Methotrexate, Leflunomide or Azathioprine in the time-periods^*^ of interest. | Patients in the denominator who did not have a value recorded for BP in the 3 months prior to the search index date. |
| 1.4 How does the DMARD monitoring rate vary across practices? | **Practice Missed Monitoring Rate:** Proportion of patients taking Methotrexate, Leflunomide or Azathioprine who were overdue at least one monitoring test, broken down by their registered GP practice. | Patients belonging to a given practice who had a prescription issued for Methotrexate, Leflunomide or Azathioprine in the time-periods^*^ of interest. | Patients in the denominator who did not have a value recorded for BP in the 3 months prior to the search index date. |
| 2.1 Were certain demographic characteristics associated with missed monitoring? | **Age:** Proportion of patients taking Methotrexate, Leflunomide or Azathioprine who were overdue at least one monitoring test, broken down by age group. | Patients who on the search index date belonged to an age group (18-29, 30-39, 40-49, 50-59, 60-69, 70-79, 80+), and had a prescription issued for Methotrexate, Leflunomide or Azathioprine in the time-periods^*^ of interest. | Patients in the denominator who did not have a value recorded for all the following in the 3 months prior to the search index date: FBC, LFT, U&E's, Blood Pressure (for Leflunomide only). |
|  | **Sex:** Proportion of patients taking Methotrexate, Leflunomide or Azathioprine who were overdue at least one monitoring test, broken down by sex category. | Patients who on the search index date were coded with a sex category (male or female) and had a prescription issued for Methotrexate, Leflunomide or Azathioprine in the time-periods^*^ of interest. | Patients in the denominator who did not have a value recorded for all the following in the 3 months prior to the search index date: FBC, LFT, U&E's, Blood Pressure (for Leflunomide only). |
|  | **Ethnicity:** Proportion of patients taking Methotrexate, Leflunomide or Azathioprine who were overdue at least one monitoring test, broken down by ethnicity group. | Patients who in the most recent search index date were coded with an ethnicity group (White, Mixed, South Asian, Black, Other, Missing), and had a prescription issued for Methotrexate, Leflunomide or Azathioprine in the time-periods^*^ of interest. | Patients in the denominator who did not have a value recorded for all the following in the 3 months prior to the search index date: FBC, LFT, U&E's, Blood Pressure (for Leflunomide only). |
|  | **Region:** Proportion of patients taking Methotrexate, Leflunomide or Azathioprine who were overdue at least one monitoring test, broken down by region. | Patients who on the search index date lived in a region (North East, North West, Yorkshire & The Humber, East Midlands, West Midlands, East, London, South East, South West), and had a prescription issued for Methotrexate, Leflunomide or Azathioprine in the time-periods^*^ of interest. | Patients in the denominator who did not have a value recorded for all the following in the 3 months prior to the search index date: FBC, LFT, U&E's, Blood Pressure (for Leflunomide only). |
|  | **Care Home Residents:** Proportion of patients in a care home taking Methotrexate, Leflunomide or Azathioprine who were overdue at least one monitoring test. | Patients who on the search index date were coded as being in a care home, and had a prescription issued for Methotrexate, Leflunomide or Azathioprine in the time-periods^*^ of interest. | Patients in the denominator who did not have had a value recorded for all the following in the 3 months prior to the search index date: FBC, LFT, U&E's, Blood Pressure (for Leflunomide only). |
|  | **Deprivation:** Proportion of patients taking Methotrexate, Leflunomide or Azathioprine who were overdue at least one monitoring test, broken down by IMD quintile. | Patients who on the search index date belonged to a particular IMD quintile, and had a prescription issued for Methotrexate, Leflunomide or Azathioprine in the time-periods^*^ of interest. | Patients in the denominator who did not have a value recorded for all the following in the 3 months prior to the search index date: FBC, LFT, U&E's, Blood Pressure (for Leflunomide only). |
|  | **Rurality:** Proportion of patients taking Methotrexate, Leflunomide or Azathioprine who were overdue at least one monitoring test, broken down by rurality classification. | Patients who on the search index date lived in an area with a particular rurality classification (1, 2, 3, 4, 5, 6, 7, 8^†^), and had a prescription issued for Methotrexate, Leflunomide or Azathioprine in the time-periods^*^ of interest. | Patients in the denominator who did not have a value recorded for all the following in the 3 months prior to the search index date: FBC, LFT, U&E's, Blood Pressure (for Leflunomide only). |
| 2.2 Were certain clinical characteristics associated with missed monitoring? | **Dementia:** Proportion of patients with dementia taking Methotrexate, Leflunomide or Azathioprine who were overdue at least one monitoring test. | Patients who on the search index date were coded with dementia, and had a prescription issued for Methotrexate, Leflunomide or Azathioprine in the time-periods^*^ of interest. | Patients in the denominator who did not have a value recorded for all the following in the 3 months prior to the search index date: FBC, LFT, U&E's, Blood Pressure (for Leflunomide only). |
|  | **Learning Disabilities:** Proportion of patients with a learning disability taking Methotrexate, Leflunomide or Azathioprine who were overdue at least one monitoring test. | Patients who on the search index date were coded with a learning disability, and had a prescription issued for Methotrexate, Leflunomide or Azathioprine in the time-periods^*^ of interest. | Patients in the denominator who did not have a value recorded for all the following in the 3 months prior to the search index date: FBC, LFT, U&E's, Blood Pressure (for Leflunomide only). |
|  | **Severe Mental Illness:** Proportion of patients with a severe mental illness taking Methotrexate, Leflunomide or Azathioprine who were overdue at least one monitoring test. | Patients who on the search index date were coded with a severe mental illness, and had a prescription issued for Methotrexate, Leflunomide or Azathioprine in the time-periods^*^ of interest. | Patients in the denominator who did not have a value recorded for all the following in the 3 months prior to the search index date: FBC, LFT, U&E's, Blood Pressure (for Leflunomide only). |

* The time-periods of interest are defined as 3 months and ii) 3-6 months prior to the search index date.

† 1 - Urban major conurbation, 2 - Urban minor conurbation, 3 - Urban city and town, 4 - Urban city and town in a sparse setting, 5 - Rural town and fringe, 6 - Rural town and fringe in a sparse setting, 7 - Rural village and dispersed, 8 - Rural village and dispersed in a sparse setting

Table S3. DMARD missed monitoring rates at baseline (Dec19 to Feb20), lockdown (Mar20 to May20) and recovery (May22 to Jul22), with cumulative counts of missed monitoring events and unique patients experiencing a missed monitoring event over the full study period (Nov19 to Jul22), broken down by medication and monitoring test.

| **Category** | **Missed Monitoring Rate** | | | | **Cumulative Count** | | | |
| --- | --- | --- | --- | --- | --- | --- | --- | --- |
|  | **Baseline Period** | **Lockdown Period** | **Recovery Period** | **Impact** (Lockdown – Baseline) | **Denominator Events** | **Number of missed monitoring events (% of denominator)** | **Number of unique patients with a missed monitoring event** | **Ratio of missed monitoring events to unique patients with a missed monitoring event** |
| Population | 28.4% | 40.8% | 28.1% | 12.4 | 3,146,849 | 977,354 (31.1%) | 117,784 | 8.3 |
| Methotrexate | 21.7% | 33.3% | 22.3% | 11.7 | 2,106,050 | 517,770 (24.6%) | 74,048 | 7.0 |
| Azathioprine | 38.5% | 50.8% | 35.5% | 12.3 | 847,125 | 333,725 (39.4%) | 34,892 | 9.6 |
| Leflunomide | 56.1% | 76.7% | 57.9% | 20.7 | 193,665 | 125,850 (65.0%) | 9,302 | 13.5 |
| FBC | 23.2% | 35.8% | 23.6% | 12.6 | 3,146,849 | 820,895 (26.1%) | 112,599 | 7.3 |
| LFT | 22.0% | 34.8% | 23.0% | 12.8 | 3,146,849 | 793,870 (25.2%) | 112,858 | 7.0 |
| U&E | 22.8% | 35.5% | 23.6% | 12.7 | 3,146,849 | 810,255 (25.7%) | 112,805 | 7.2 |
| BP | 45.7% | 70.2% | 49.9% | 24.5 | 193,665 | 111,215 (57.4%) | 9,008 | 12.3 |

Table S4. DMARD missed monitoring rates* for the population broken down into demographic and clinical characteristics at baseline (Dec19 to Feb20), lockdown (Mar20 to May20) and recovery (May22 to Jul22).

| **Characteristic** | **Category** | **Missed Monitoring Rate**  (%) | | | **Impact Significance**  (percentage points) | | | | | **Subgroup Heterogeneity** | |
| --- | --- | --- | --- | --- | --- | --- | --- | --- | --- | --- | --- |
|  |  | **Baseline** | **Lockdown** | **Recovery** | **Impact** (Lockdown – Baseline) | **95% CI Lower Limit** | **95% CI Upper Limit** | **t-value** | **p-value** | **Cochran's Q** | **p-value** |
| Population | Not Applicable | 28.4% | 40.8% | 28.1% | 12.4 | 12.0 | 12.8 | 57.49 | <0.001 | N/A | N/A |
| Age Band | 18-29 | 44.5% | 52.8% | 42.9% | 8.3 | 6.1 | 10.5 | 7.26 | <0.001 | 37.526 | <0.001 |
|  | 30-39 | 39.1% | 49.7% | 38.5% | 10.6 | 8.8 | 12.3 | 11.86 | <0.001 |  |  |
|  | 40-49 | 34.8% | 45.4% | 35.2% | 10.6 | 9.2 | 11.9 | 15.57 | <0.001 |  |  |
|  | 50-59 | 31.0% | 43.4% | 30.7% | 12.4 | 11.4 | 13.4 | 25.00 | <0.001 |  |  |
|  | 60-69 | 26.0% | 38.7% | 26.2% | 12.7 | 11.9 | 13.6 | 29.06 | <0.001 |  |  |
|  | 70-79 | 22.5% | 36.2% | 22.9% | 13.7 | 12.9 | 14.5 | 32.64 | <0.001 |  |  |
|  | 80+ | 23.3% | 36.5% | 22.4% | 13.2 | 12.0 | 14.4 | 21.77 | <0.001 |  |  |
| Sex | Female | 27.6% | 40.4% | 27.5% | 12.8 | 12.2 | 13.3 | 46.27 | <0.001 | 4.500 | 0.034 |
|  | Male | 29.5% | 41.3% | 28.9% | 11.9 | 11.2 | 12.5 | 34.24 | <0.001 |  |  |
| Ethnicity | Black | 35.5% | 47.2% | 34.8% | 11.7 | 7.0 | 15.6 | 5.12 | <0.001 | 10.071 | 0.073 |
|  | Missing | 30.2% | 44.7% | 28.5% | 14.5 | 8.9 | 18.9 | 5.44 | <0.001 |  |  |
|  | Mixed | 35.4% | 50.0% | 36.0% | 14.6 | 8.5 | 19.8 | 4.90 | <0.001 |  |  |
|  | Other | 33.1% | 47.1% | 34.0% | 14.0 | 8.9 | 19.2 | 5.34 | <0.001 |  |  |
|  | South Asian | 39.2% | 54.4% | 36.8% | 15.2 | 13.2 | 17.1 | 15.17 | <0.001 |  |  |
|  | White | 27.6% | 39.8% | 27.3% | 12.2 | 11.8 | 12.7 | 54.62 | <0.001 |  |  |
| Region | East | 31.2% | 43.7% | 30.6% | 12.5 | 11.6 | 13.3 | 28.66 | <0.001 | 78.869 | <0.001 |
|  | East Midlands | 26.5% | 37.7% | 28.8% | 11.2 | 10.2 | 12.2 | 21.63 | <0.001 |  |  |
|  | London | 46.5% | 58.1% | 38.7% | 11.6 | 9.0 | 14.0 | 8.97 | <0.001 |  |  |
|  | North East | 38.0% | 47.3% | 35.4% | 9.2 | 7.3 | 11.2 | 9.35 | <0.001 |  |  |
|  | North West | 20.5% | 37.5% | 20.0% | 17.0 | 15.6 | 18.4 | 23.88 | <0.001 |  |  |
|  | South East | 30.6% | 43.2% | 32.3% | 12.7 | 11.0 | 14.3 | 15.23 | <0.001 |  |  |
|  | South West | 25.0% | 37.5% | 24.1% | 12.4 | 11.4 | 13.5 | 23.52 | <0.001 |  |  |
|  | West Midlands | 28.7% | 45.3% | 29.6% | 16.6 | 14.3 | 19.0 | 13.75 | <0.001 |  |  |
|  | Yorkshire and The Humber | 25.1% | 36.6% | 24.6% | 11.5 | 10.4 | 12.5 | 21.20 | <0.001 |  |  |
| Care Home | TRUE | 29.4% | 41.8% | 27.0% | 12.5 | 8.9 | 16.7 | 6.40 | <0.001 | 0.040 | 0.842 |
|  | FALSE | 28.3% | 40.7% | 28.1% | 12.4 | 12.0 | 12.8 | 57.14 | <0.001 |  |  |
| Dementia | TRUE | 29.3% | 43.0% | 27.0% | 13.7 | 10.3 | 17.4 | 7.66 | <0.001 | 0.598 | 0.44 |
|  | FALSE | 28.3% | 40.7% | 28.1% | 12.4 | 12.0 | 12.8 | 56.99 | <0.001 |  |  |
| Housebound | TRUE | 29.1% | 38.7% | 27.7% | 9.6 | 6.8 | 12.8 | 6.46 | <0.001 | 3.183 | 0.074 |
|  | FALSE | 28.3% | 40.8% | 28.1% | 12.5 | 12.1 | 12.9 | 57.10 | <0.001 |  |  |
| Learning Disability | TRUE | 32.4% | 49.3% | 36.0% | 16.9 | 9.1 | 23.7 | 4.41 | <0.001 | 1.165 | 0.28 |
|  | FALSE | 28.3% | 40.7% | 28.0% | 12.4 | 12.0 | 12.8 | 57.33 | <0.001 |  |  |
| Serious Mental Illness | TRUE | 29.2% | 40.9% | 30.9% | 11.7 | 7.6 | 16.2 | 5.40 | <0.001 | 0.051 | 0.821 |
|  | FALSE | 28.3% | 40.8% | 28.0% | 12.4 | 12.0 | 12.8 | 57.24 | <0.001 |  |  |
| Deprivation Score | 1st quintile (most deprived) | 30.6% | 43.2% | 29.7% | 12.6 | 11.6 | 13.7 | 23.18 | <0.001 | 3.152 | 0.533 |
|  | 2nd quintile | 29.2% | 42.1% | 29.0% | 12.9 | 11.9 | 13.9 | 25.61 | <0.001 |  |  |
|  | 3rd quintile | 28.5% | 40.9% | 27.7% | 12.4 | 11.5 | 13.3 | 27.01 | <0.001 |  |  |
|  | 4th quintile | 27.4% | 39.9% | 27.4% | 12.5 | 11.6 | 13.4 | 27.56 | <0.001 |  |  |
|  | 5th quintile (least deprived) | 26.7% | 38.4% | 27.0% | 11.7 | 10.8 | 12.6 | 25.06 | <0.001 |  |  |
| Rural-Urban Classification | 1 (most urban) | 32.4% | 45.5% | 30.5% | 13.1 | 12.0 | 14.2 | 23.34 | <0.001 | 13.323 | 0.065 |
|  | 2 | 30.2% | 40.0% | 29.3% | 9.8 | 8.1 | 11.5 | 11.34 | <0.001 |  |  |
|  | 3 | 29.3% | 41.9% | 29.5% | 12.6 | 12.0 | 13.2 | 42.16 | <0.001 |  |  |
|  | 4 | 19.1% | 28.3% | 10.6% | 9.1 | 1.0 | 16.5 | 2.21 | 0.027 |  |  |
|  | 5 | 23.4% | 35.6% | 23.7% | 12.2 | 11.1 | 13.3 | 21.89 | <0.001 |  |  |
|  | 6 | 16.2% | 30.8% | 16.7% | 14.6 | 10.0 | 19.1 | 6.27 | <0.001 |  |  |
|  | 7 | 25.0% | 36.8% | 24.3% | 11.8 | 10.5 | 13.1 | 17.82 | <0.001 |  |  |
|  | 8 (most rural) | 15.3% | 29.4% | 18.0% | 14.1 | 10.0 | 18.0 | 6.87 | <0.001 |  |  |

*Subgroup rates at baseline, lockdown and recovery were calculated from patient counts rounded to nearest 5 to ensure anonymity, whilst statistical tests were conducted using unrounded counts for accuracy.
